# Targeting the proteasome in cancer therapy: development and future opportunities in natural products

**DOI:** 10.3389/fphar.2026.1806787

**Published:** 2026-05-04

**Authors:** Xiu Zhao, Shanshan Liu, Xinrui Zeng, Yu Liao, Mao Zhang, Qiang Wang, Dan Zhang, Qifeng Chen, Miao Xian, Yong Qin

**Affiliations:** 1 Key Laboratory of Drug-Targeting and Drug Delivery System of the Education Ministry and Sichuan Province, Sichuan Engineering Laboratory for Plant-Sourced Drug, West China School of Pharmacy, Sichuan University, Chengdu, China; 2 Center for Translational Research in Hematological Malignancies, Houston Methodist Neal Cancer Center, Houston Methodist Academic Institute, Houston, TX, United States

**Keywords:** cancer therapy, drug resistance, multiple myeloma, natural product, proteasome inhibitor

## Abstract

**Background:**

The ubiquitin–proteasome pathway is a critical therapeutic target in malignancies, particularly multiple myeloma. The development of proteasome inhibitors (PIs) has marked a milestone in multiple myeloma therapy and now constitutes the backbone of frontline treatment regimens. However, primary and acquired resistance remain a major challenge, underscoring the need to identify new PIs as well as agents that can resensitize resistant cells.

**Method:**

This review provides an overview of the current clinical applications of PIs and offers a comprehensive summary of natural products that either directly target the proteasome or enhance cellular sensitivity to PIs. Relevant clinical trials and literature published up to 2025 were retrieved from PubMed, Web of Science, Google Scholar, and ClinicalTrials.gov, focusing on the biological and pharmacological activities, structure-activity relationships, and clinical outcomes.

**Results:**

In this review, we summarize the current clinical applications of PIs and specifically highlight the discovery of natural products that directly target the proteasome or enhance cellular sensitivity to PIs. We also discuss the clinical progress of marizomib, the only natural product-derived PI, that has advanced to clinical trials. Despite existing challenges, natural product-derived PIs hold significant potential for the development of next-generation therapies that can overcome resistance and improve clinical outcomes.

**Conclusion:**

The development of novel natural product-derived PIs and rational combination strategies offers promising opportunities for overcoming resistance in cancer therapy. Although challenges remain, the remarkable structural diversity of natural products provides a rich reservoir for drug discovery, underscoring the importance of continued exploration and innovation in this field.

## Graphical Abstract

**Figure:**
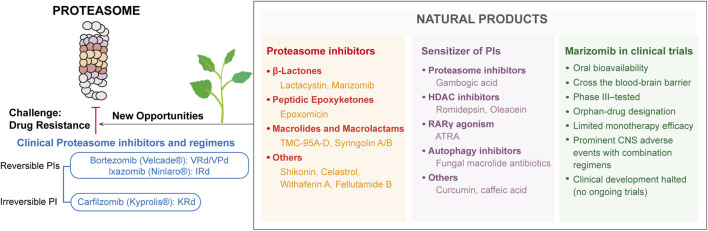

## 1 Introduction

The ubiquitin-proteasome pathway (U*PP*) is a highly complex protein degradation system that universally exists in eukaryotic cells (Schwartz and Ciechanover, 2009). As one of the most crucial regulated protein degradation mechanisms, the U*PP* controls various key biological processes, including apoptosis, cell cycle, cell survival, DNA repair, and antigen presentation (Zhang Y. W. et al., 2022; Wang Q. Q. et al., 2020; Luo et al., 2023). Its proper function relies on the precise coordination of several steps, such as polyubiquitination, deubiquitination, and the eventual degradation of target proteins, ensuring the timely removal of damaged, misfolded, or unnecessary proteins (Manasanch and Orlowski, 2017).

**FIGURE 1:**
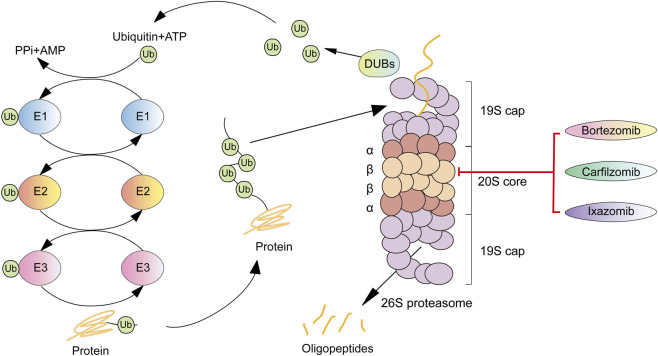
Schematic illustration of the ubiquitin-proteasome system. Ubiquitin is sequentially transferred through E1, E2, and E3 enzymes and conjugated to substrate proteins. Ubiquitinated substrates are subsequently deubiquitinated by deubiquitinases (DUBs) and degraded by the proteasome. Proteasome inhibitors inhibit proteasome activity by targeting the catalytic β subunits of the 20S core particle.

The 26S proteasome consists of a 20S catalytic core particle and a 19S or 11S regulatory particle. The 20S core contains three catalytic sites: caspase-like (C-L, β1), trypsin-like (T-L, β2), and chymotrypsin-like (CT-L, β5). Among these, the chymotrypsin-like activity of the β5 subunit serves as the primary target for most proteasome inhibitors (PIs) (Figure 1). By disrupting the U*PP*, PIs block protein degradation—a pathway upon which tumor cells are particularly dependent—resulting in the accumulation of misfolded proteins, induction of endoplasmic reticulum stress, and activation of apoptotic pathways, thereby suppressing tumor growth (Hoeller and Dikic, 2009; Wang and Liu, 2022). Current evidence indicates that FDA-approved PIs can achieve suppression of proteasomal activity while demonstrating antitumor effects in clinical applications, particularly showing outstanding efficacy in multiple myeloma (MM) (Esseltine and Mulligan, 2012; Manasanch and Orlowski, 2017). Since the approval of the first proteasome inhibitor, bortezomib, by the FDA in 2003 for the treatment of MM, PIs have become the therapeutic backbone of MM therapy and have significantly prolonged patient survival (Li J. et al., 2025). Bortezomib in combination with lenalidomide and dexamethasone (VRd) has been established as a standard frontline regimen for patients with newly diagnosed multiple myeloma (NDMM) (Callander et al., 2022; Shen et al., 2023). However, as with nearly all cancer therapies, both acquired and intrinsic resistance are the major challenges to the effectiveness of PIs. Although multiple mechanisms underlying the resistance have been identified, overcoming proteasome inhibitor resistance remains an unresolved challenge and requires further investigation (Manasanch and Orlowski, 2017; Nikesitch and Ling, 2016; Tang et al., 2023).

Natural products derived from plants and marine organisms represent a valuable source for target finding, mechanism investigation, and drug discovery (Jia et al., 2024; An et al., 2024; Cai et al., 2023; Peng et al., 2022; Peng et al., 2020a; Peng et al., 2020b). Over the past few decades, numerous clinically important drugs—such as vincristine (Wang et al., 2016; Zhang et al., 2017), irinotecan (Wu et al., 2020; Hou et al., 2025; Lin et al., 2020), homoharringtonine (Liu et al., 2023; Wang F. F. et al., 2021), galantamine (Zhang et al., 2023b), paclitaxel (Taxol) (Duan et al., 2024; Hou et al., 2025; Lin et al., 2020), and artemisinin (Li X. Y. et al., 2018; Wang Y. et al., 2020; Zhu et al., 2021)—have been discovered or developed from natural products. Furthermore, several natural products, including lactacystin (Delic et al., 1998), epoxomicin (Meng et al., 1999), celastrol (Yang et al., 2006), and marizomib (Ma and Diao, 2015), have been reported to exhibit proteasome-inhibitory activity or to enhance the cytotoxic effects of established PIs.

In this review, we summarize the development of PIs, with a particular focus on those derived from natural products, as well as natural products that potentiate the activity of PIs in preclinical and clinical settings.

## 2 Overview of proteasome inhibitors

### 2.1 Development of proteasome inhibitors

**FIGURE 2:**
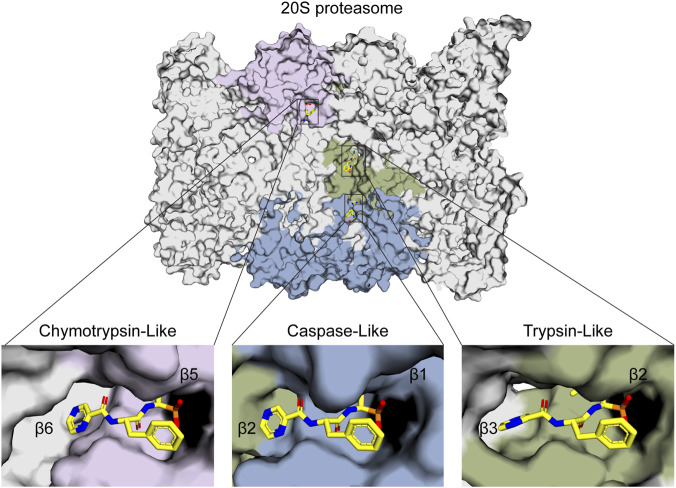
Structure of the 20S proteasome-bortezomib complex. A cross-section of the proteasome is shown for clarity. Bortezomib binds to the β1/β2/β5 subunits. The inset shows a surface representation of the binding between bortezomib and the active pockets. Bortezomib is colored yellow, and the β1 (pale blue), β2 (beige-green), and β5 (lilac) subunits are indicated accordingly. Thr1 residues that form covalent bonds with the boronic acid group of bortezomib, thereby inhibiting proteasome catalytic activity, are colored black. (PDB: 5LF3).

The development of PIs has been markedly optimized from the first to the second generation. Bortezomib (BTZ, Velcade®), a purely synthetic small molecule and the first FDA-approved proteasome inhibitor, exerts its antitumor activity by reversibly inhibiting the chymotrypsin-like activity of the β5 subunit of the proteasome. BTZ demonstrated remarkable efficacy in early clinical trials, leading to its FDA approval in 2003 for relapsed or refractory MM and in 2008 for newly diagnosed disease (Orlowski et al., 2002; Richardson et al., 2006). Today, BTZ is established as a first-line therapy and remains a cornerstone in the management of this disease. The proteasome-BTZ complex structure is shown (Figure 2), highlighting bortezomib-targeted subunits and inhibitor-binding clefts. Based on the structural insights into proteasome-inhibitor interactions and the clinical success of BTZ, efforts were made to develop second-generation PIs with improved efficacy and safety profiles. Building upon this, the second-generation inhibitor carfilzomib (CFZ, Kyprolis®), which is a fully synthetic inhibitor inspired by natural scaffolds, made a significant breakthrough: unlike the reversible boronate binding of BTZ, CFZ irreversibly binds to the N-terminal threonine of the proteasome via its epoxyketone pharmacophore. Compared with BTZ, CFZ is associated with a markedly lower incidence of peripheral neuropathy, which can be attributed to its higher specificity for the proteasomal β5 subunit and its faster metabolic elimination (O'Connor et al., 2009; Alsina et al., 2012). The clinical development of ixazomib (Ninlaro®), another purely synthetic agent optimized from bortezomib scaffolds and the first oral proteasome inhibitor approved by the FDA in 2015 (Shirley, 2016), has further improved the treatment of MM patients by offering a therapy more suitable for long-term maintenance and improving patient adherence. Ixazomib is associated with lower cardiotoxicity compared with carfilzomib (Lee et al., 2025) and reduced neurotoxicity compared with bortezomib (Sana et al., 2020).

PIs now constitute the backbone of numerous regimens for MM that integrate immunomodulatory drugs, glucocorticoids, monoclonal antibodies, and alkylating agents (Jiang et al., 2022; Wang F. R. et al., 2025; Yu et al., 2026; Nijhof et al., 2018). The most established combination regimen for the clinical treatment of MM consists of a proteasome inhibitor (PI), an immunomodulatory drug (IMiD), and dexamethasone (Dex). In recent years, the emergence of the anti-CD38 antibody daratumumab (Leleu et al., 2022) has led to the development of a series of regimens incorporating daratumumab into existing therapeutic backbones, further improving clinical efficacy. The key features of the three FDA-approved proteasome inhibitors and their representative clinical combination regimens for MM treatment are summarized in Table 1.

**TABLE 1:** Development of FDA-approved proteasome inhibitors.

PIs	Structural formula	Target (EC50 in MM cells)	Characteristics	Representative clinical regimens
Bortezomib (Velcade®,approved in 2003)	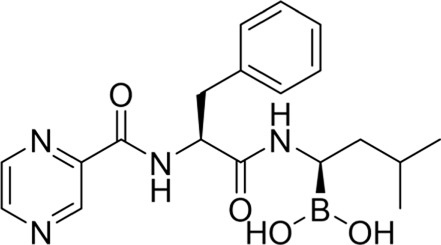	CT-L: 13.83 nM T-L: >10,000 nM C-L: 41.39 nM (Besse et al., 2017)	1. First approved PI 2. Dose-limiting toxicity Peripheral neuropathy 3. VRD regimen serves as the standard first-line treatment for NDMM 4. Administration: intravenous or subcutaneous injection	Bortezomib and Dexamethasone (Vd) (NCT00335348, NCT00391157, NCT00401011)
				Bortezomib, Cyclophosphamide, and Dexamethasone (VCd) (NCT00609167, NCT00813150, NCT01971658, NCT00833560)
				Bortezomib, Lenalidomide/Pomalidomide, and Dexamethasone (VRd/VPd) (VRd: NCT01191060, NCT01916252, NCT01782963 VPd: NCT01734928, NCT01212952)
				Daratumumab, Bortezomib, Lenalidomide + Dexamethasone (Dara-VRd) (NCT02874742)
Carfilzomib (Kyprolis®,approved in 2012)	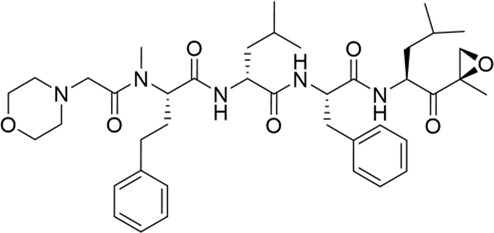	CT-L: 136 nM T-L: 2363 nM C-L: 5954 nM (Besse et al., 2017)	1. Rapid elimination and enhanced proteasome selectivity, reducing off-target toxicity 2. Significantly lower incidence of peripheral neuropathy compared to bortezomib 3. Cardiovascular toxicity (e.g., hypertension, heart failure) requires monitoring 4. Administration: Intravenous injection	Carfilzomib, and Dexamethasone (Kd)
				Carfilzomib, Lenalidomide/Pomalidomide, and Dexamethasone (KRd/KPd) (KRd: NCT03375567, NCT04065789 KPd: NCT04191616, NCT02185820, NCT01665794)
				Daratumumab, Carfilzomib, Lenalidomide + Dexamethasone (Dara-KRd) (NCT04065789, NCT03224507, NCT01998971)
Ixazomib (Ninlaro®,approved in 2015)	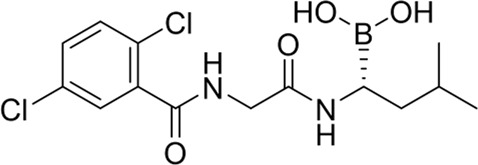	CT-L: 3012 nM T-L: >>10,000 nM C-L: 624 nM (Besse et al., 2017)	1. First approved oral PI 2. Main adverse effects include gastrointestinal reactions (diarrhea, nausea), rash, and grade 3–4 thrombocytopenia 3. no significant peripheral neuropathy 4. Administration route: Oral	Ixazomib, Lenalidomide, and Dexamethasone (IRd) (NCT01564537)

### 2.2 Drug resistance and related mechanisms of proteasome inhibitors

Despite the application of PIs brings great benefit for MM patients, most patients will relapse and finally become resistant to former regimens. Notably, proteasome inhibition acutely activates multiple adaptive resistance pathways, thereby diminishing the therapeutic effectiveness of PIs.

#### 2.2.1 Proteasome-dependent resistance mechanisms

**FIGURE 3:**
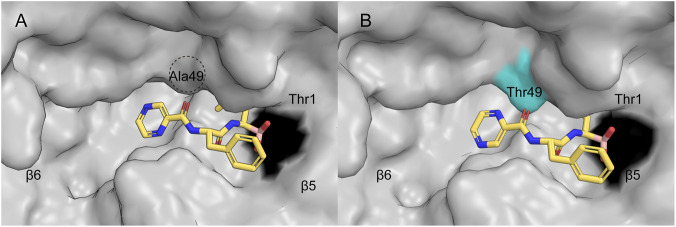
The wild-type/A49T-mutant proteasome β5 subunit bound to BTZ. Surface representations illustrate the β5 substrate binding channel with bound BTZ in wild-type **(A)** and mutant **(B)** yeast proteasome core particles (yCPs). Cyan coloring indicates mutations, black denotes the active site Thr1, and dotted circles highlight Ala49 in the wild-type structure. (PDB: 4QVL, 4QVY).

Studies have revealed that tumor cells may develop resistance through alterations in the proteasome itself. For instance, a potential mechanism underlying acquired resistance to BTZ may involve mutations (e.g.,: A49T, Figure 3) within the binding pocket of the proteasome β5 subunit or upregulation of its expression level (Lü et al., 2008; Oerlemans et al., 2008). However, sequencing of PSMB5 gene in MM patients treated with BTZ did not show a relationship between single nucleotide polymorphisms in PSMB5 and response to BTZ, which may be attributed to the limited sample size (Wang et al., 2008). These findings suggest that direct genetic alterations in the proteasome may not represent the dominant mechanism of resistance in clinical settings and that additional adaptive mechanisms are likely involved.

#### 2.2.2 Role of immunoproteasome composition in PI sensitivity and resistance

Multiple myeloma exhibits exceptional sensitivity to proteasome inhibitors (PIs), which is partly attributed to the high abundance of the immunoproteasome in plasma cells. The immunoproteasome is a specialized variant of the 20S proteasome in which the constitutive catalytic subunits β1 (PSMB6), β2 (PSMB7), and β5 (PSMB5) are replaced by inducible counterparts β1i (PSMB9), β2i (PSMB10), and β5i (PSMB8). This complex is highly expressed in immune and hematopoietic cells due to their increased demand for protein turnover during processes such as immunoglobulin production and antigen presentation (Zhang Y. W. et al., 2022).

The relative abundance of immunoproteasome versus constitutive proteasome has been reported to correlate with cellular sensitivity to PIs. For example, a higher immunoproteasome-to-constitutive proteasome ratio has been associated with significantly increased sensitivity to proteasome inhibition, with acute lymphoblastic leukemia cells showing up to 5.5-fold greater sensitivity to PIs than acute myeloid leukemia cells (Niewerth et al., 2013).

Proteasome subunit switching can also contribute to the development of drug resistance. In both myeloma and AML cells, a near-complete shift from the immunoproteasome subunit β5i to the constitutive β5 subunit has been observed in bortezomib-resistant cells (Franke et al., 2012).

#### 2.2.3 Adaptive proteostasis rewiring

Beyond direct alterations in the proteasome, tumor cells can develop adaptive resistance to proteasome inhibitors (PIs) through adaptive proteostasis rewiring (Sedlacek, 2025). This process involves multiple compensatory mechanisms that help cells alleviate the accumulation of misfolded or damaged proteins induced by proteasome inhibition.

One important adaptive pathway is the autophagy-lysosome system, which can serve as an alternative protein degradation mechanism when proteasome activity is inhibited (Dong et al., 2023; Fu et al., 2025; Xiang H. G. et al., 2020). In a phase II clinical trial, the histone deacetylase inhibitor panobinostat, which inhibits aggresome formation, was combined with BTZ and dexamethasone for the treatment of patients with RRMM following prior BTZ therapy, and demonstrated clinical activity (Richardson et al., 2013). Among the 55 patients enrolled in this study, 34.5% achieved a partial response or better, and an additional 18% of patients experienced a reduction in tumor burden of approximately 25%–50%.

Another key response involves the heat-shock response, characterized by increased expression of molecular chaperones such as HSP70 and HSP90 (Bai et al., 2023; Wang et al., 2024; Xu et al., 2023). These chaperones help maintain protein folding homeostasis and protect tumor cells from proteotoxic stress, thereby contributing to drug resistance (Lianos et al., 2015). Numerous preclinical studies have demonstrated that inhibiting various heat shock proteins, particularly HSP90, can enhance the anti-tumor efficacy of PIs (Mitsiades et al., 2006; Mimnaugh et al., 2004; Ishii et al., 2012). Early-phase clinical trials have also confirmed that the combination of HSP90 inhibitor KW-2478 and BTZ was well tolerated in RRMM patients (Cavenagh et al., 2017). Though no randomized studies have demonstrated that such combination regimens are superior in efficacy to existing standard therapies, further exploration of alternate dosing schedules and combinations would be supported by the good tolerability.

Proteasome inhibition can also activate the unfolded protein response (UPR) of ER stress (Huang et al., 2020; Li X. B. et al., 2025; Meng et al., 2024), a cellular stress response that enhances the capacity of the endoplasmic reticulum to process misfolded proteins and restore proteostasis.

In summary, despite significant progress in elucidating the mechanisms underlying PI resistance, clinically effective strategies to overcome this limitation are still under investigation. Further exploration is needed to guide the development of next-generation therapies.

## 3 Natural product-derived proteasome inhibitors

Natural product-derived molecules, widely distributed across plants, microorganisms, and marine organisms, are characterized by remarkable structural diversity and represent a valuable reservoir for drug discovery (Cui et al., 2023; Kim et al., 2023). Unlike synthetic compounds, natural products possess unique structural scaffolds and inherent biocompatibility shaped by biological evolution, enabling them to exert functions that are often difficult to achieve through chemical synthesis (Wijayasinghe et al., 2021; Zhang et al., 2024). The discovery of novel PIs from natural sources holds great promise for overcoming drug resistance and enhancing therapeutic efficacy. In this section, we summarize representative natural PIs by scaffold, together with their mechanisms of action (Table 2).

**TABLE 2:** Natural product-derived proteasome inhibitors.

Classes of natural products	Name	Source	Proteasome inhibition	Related mechanisms
β-Lactones	Lactacystin	*Streptomyces spp* (Omura et al., 1991)	*k*_assoc_ (human 20S proteasome) CT-L: 194 ± 15 M^-1^s^-1^ (10 μM) T-L: 10.1 ± 1.8 M^-1^s^-1^ (10 μM) C-L: 4.2 ± 0.6 M^-1^s^-1^ (100 μM) (Fenteany et al., 1995)	NF-κB suppression, IκBα accumulation (Delic et al., 1998; Shirley et al., 2005; Li et al., 2015b) NOXA induction, apoptosis (Qin et al., 2005) ↑PDI, GRP78, CHOP, cleaved caspases (Qin et al., 2005; Xu et al., 2015)
	Marizomib	*Salinispora tropica* (Feling et al., 2003)	IC50 (human 20S proteasome) CT-L: 18.5 nM T-L: 326.5 nM C-L: 596.6 nM (Sülzen et al., 2025)	p27 and p21 accumulation, apoptosis (Miller et al., 2011; Miller et al., 2007) ↑GRP78, CHOP, ATF4 (Manton et al., 2016)
Peptidic Epoxyketones	Epoxomicin	actinomycete strain Q996-17 (Hanada et al., 1992)	k_obs_/[I] (bovine 20S proteasome) CT-L: 20,000 ± 3,000 M-^1^s^-1^ (0.037–0.1 μM) T-L: 43 ± 10 M^-1^s^-1^ (25–75 μM) C-L: 310 ± 70 M^-1^s^-1^ (0.25–2.5 μM) (Meng et al., 1999)	TNF-α-induced IκBα, DNA-binding inhibition (Meng et al., 1999), NF-κB and IRF3 suppression (Kim et al., 2021)
Macrolides and Macrolactams	TMC-95A–D	*Apiospora montagnei Sacc.* TC 1093 (Koguchi et al., 2000)	IC_50_ (human 20S proteasome, TMC-95A) CT-L: 5.4 nM T-L: 200 nM C-L: 60 nM (Koguchi et al., 2000)	N/A
	Syringolin A/B	*Oryza sativa* L. (Wäspi et al., 1998; Wäspi et al., 1999)	*k*_assoc_ (human 20S proteasome, Syringolin A) CT-L: 863 ± 106 M^-1^s^-1^ (100–200 nM) T-L: 94 ± 12 M^-1^s^-1^ (150–600 nM) C-L: 6 ± 0.3 M^-1^s^-1^ (20–40 μM) (Groll et al., 2008b; Clerc et al., 2009a)	N/A
Other types	Shikonin	*Lithospermum erythrorhizon* Siebold and Zucc *, Arnebia euchroma* I.M.Johnst. (Yang et al., 2009)	IC_50_ (rabbit 20S proteasome) CT-L: 12.5 μM (Yang et al., 2009)	Accumulation of pro-apoptotic proteins (IκBɑ, Bax, p27); degradation of cIAP1/2, RIP1; ER stress (Lu et al., 2011)
	Celastrol and pristimerin	*Tripterygium wilfordii* Hook.f. (Dai et al., 2023)	IC_50_ (rabbit 20S proteasome) CT-L: 2.2 μM (Yang et al., 2008)	Accumulation of IκBɑ, Bax, and p27; ubiquitinated proteins blockade, NF-κB suppression, androgen receptor downregulation (Yang et al., 2008; Yang et al., 2006)
	Withaferin A	*Withania somnifera* (L.) Dunal	IC_50_ (rabbit 20S proteasome) CT-L: 4.5 μM (Yang et al., 2007)	Ubiquitinated proteins blockade (Yang et al., 2007; Yang et al., 2012; Xing et al., 2023), ↑BiP, GRP94, HSP70, HSP30, IDH1 (Khan et al., 2012; Xu et al., 2019)
	Fellutamide B	*Penicillium fellutanum* (Hines et al., 2008)	IC_50_ (bovine 20S proteasome) CT-L: 9.4 ± 2.5 nM T-L: 2.0 ± 0.4 µM C-L: 1.2 ± 0.8 µM (Hines et al., 2008; Lin et al., 2010)	↑NGF (Hines et al., 2008)

### 3.1 β-Lactones

#### 3.1.1 Lactacystin

**FIGURE 4:**
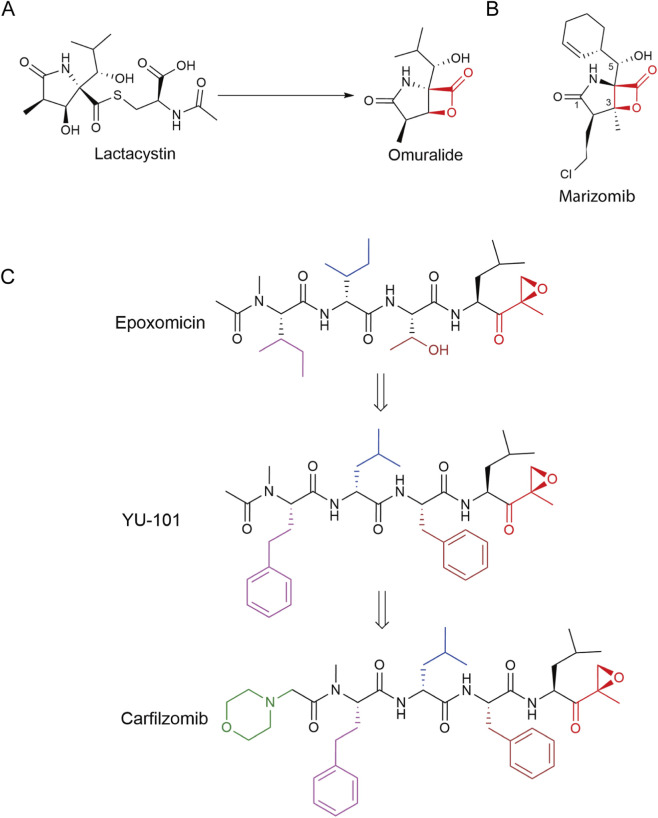
Structures of β-lactone natural product proteasome inhibitors. **(A)** Structure of lactacystin and the bioactive metabolite clasto-lactacystin-β-lactone (omuralide) was demonstrated. **(B)** Structure of marizomib, isolated from the marine actinomycete *Salinispora tropica,* was demonstrated. **(C)** Structures of epoxomicin, YU-101, and carfilzomib were demonstrated. The regions of structural modification in each compound are highlighted in different colors. Compound warhead labeled in red.

Lactacystin (Figure 4A) is a natural proteasome inhibitor isolated from *Streptomyces spp*. (Omura et al., 1991) that functions *in vivo* as a pro-drug, generating the bioactive metabolite clasto-lactacystin-β-lactone (omuralide) (Dick et al., 1997). Omuralide covalently modifies the β5 (PSMB5/PRE2) subunit responsible for the CT-L (chymotrypsin-like) activity of the 20S proteasome (Groll et al., 1997), thereby reversibly inhibiting its proteolytic function while weakly and reversibly affecting T-L (trypsin-like) and C-L (caspase-like) activities (Fenteany et al., 1995).

**FIGURE 5:**
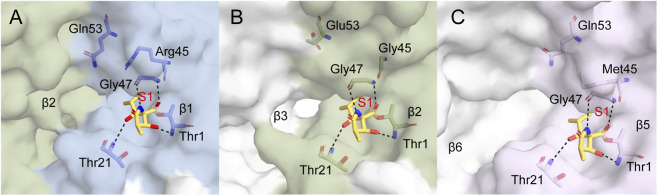
20S proteasome in complex with omuralide. Binding mode of omuralide (yellow) to the β1 (pale blue) **(A)**, β2 (beige-green) **(B)**, and β5 (lilac) **(C)** subunits. Interacting residues are shown as sticks; hydrogen bonds are depicted as black dashed lines. (PDB: 4QZZ).

Upon binding to the β1, β2, and β5 subunits, the β-lactone ring of omuralide undergoes nucleophilic attack by the Thr1Oγ residue located within the S1 substrate-binding pocket of each subunit, resulting in ring opening and the formation of a slowly reversible covalent acyl-enzyme intermediate (Groll et al., 2006b). Concurrently, hydrogen bonding interactions are established with Thr1, Thr21, and Gly47 (Groll et al., 2008a). However, the S1 pocket of β5 exhibits a hydrophobic character that exhibits favorable complementarity with the isopropyl and alkyl side chains of omuralide’s hydrophobic scaffold. In contrast, the Glu53 residue at the base of the β2-S1 pocket and the Arg45 residue at the base of the β1-S1 pocket both carry electrostatic charges, which generate repulsive forces against these hydrophobic moieties. Consequently, omuralide fails to achieve stable residence within the S1 pockets of β1 and β2 subunits necessary for the completion of the covalent reaction, accounting for the negligible inhibitory potency of omuralide toward the β1 and β2 subunits (Figure 5).

Lactacystin exhibits broad anti-tumor activity, inducing apoptosis in diverse tumor cell lines while sparing normal cells. The apoptotic mechanisms of lactacystin are cell-context dependent. In leukemic cells, lactacystin-induced apoptosis correlated with decreased nuclear expression of the p50 and p65 subunits of NF-κB and elevated cytoplasmic IκBα (Delic et al., 1998). In melanoma, lactacystin upregulated NOXA, leading to cleavage of caspases-3, -8, -9, and PARP, thereby initiating a mitochondrion-dependent apoptotic cascade (Qin et al., 2005). In LNCaP prostate cancer cells, it suppressed p50, enhanced caspase-3 cleavage, and elevated phosphorylated IκBα (Shirley et al., 2005), whereas in gastric cancer models it reduced NF-κB activity in MKN28 but not SGC7901 cells (Li Y. et al., 2015). In HeLa cells, lactacystin synergized with cisplatin, amplifying ER stress–mediated apoptosis via upregulation of PDI, GRP78, CHOP, and cleaved caspases (Xu et al., 2015), while down-regulating hCTR1, reducing cisplatin uptake, and overcoming resistance (Holzer and Howell, 2006).

Beyond cytotoxicity, lactacystin also modulates immune responses by attenuating MHC class I antigen presentation. By inhibiting 20S β-subunits, it reduced generation of antigenic peptides, suppressing the presentation of ovalbumin-derived (Craiu et al., 1997) and viral epitopes (e.g., NP366–374, gp33, gp276) (Cerundolo et al., 1997). As it irreversibly alkylated the active-site threonine of β5, inhibition persists after drug removal (Gallimore et al., 1998). Interestingly, low non-toxic doses (0.5–1 µM) of lactacystin selectively inhibited CT-L activity and reshaped proteasomal cleavage patterns: presentation of gp33, NP118, and pp89-168 was diminished, whereas gp276 presentation was enhanced (Schwarz et al., 2000). This selective modulation of proteasome processing warrants further investigation and may suggest potential applications in autoimmunity, transplantation, and tumor immunotherapy.

#### 3.1.2 Marizomib

Marizomib (MRZ, salinosporamide A, NPI-0052; Figure 4B) was first isolated in 2003 from the marine actinomycete *Salinispora tropica.* (Feling et al., 2003). It irreversibly alkylated the N-terminal threonine (Thr1Oγ) of 20S catalytic subunits, binding all three catalytic β-subunits with the highest affinity and inhibitory efficiency toward β5 (IC_50_ = 18.5 nM), with moderate inhibitory activity also observed against the β2 and β1 subunits (β2: IC_50_ = 326.5 nM, β1: IC_50_ = 596.6 nM) (Groll et al., 2006b; Mitsiades et al., 2006; Sülzen et al., 2025).

Similar to omuralide, the nucleophilic Thr1Oγ at the N-terminus of the proteasome subunit initiates ring opening of the β-lactone moiety via nucleophilic attack, resulting in the formation of a stable covalent ester linkage between the inhibitor and the catalytic Thr1 residue. However, subsequent mechanistic events diverge from those of omuralide: the exposed C3 hydroxyl group generated upon ring opening serves as a novel nucleophilic center, which undergoes intramolecular nucleophilic attack on the C2 chloroethyl side chain. This intramolecular displacement eliminates chloride ion and concomitantly forms a five-membered tetrahydrofuran ring, thereby achieving complete protonation of the Thr1 N-terminus and effecting irreversible inhibition of proteasomal catalytic activity (Supplementary Figure S1A-C) (Groll et al., 2006b). MRZ incorporates a bulkier and more hydrophobic cyclohexenyl moiety in place of the isopropyl substituent at the C5 position found in omuralide. Consequently, NPI-0052 is similarly repelled by the electrostatically charged β1-S1 and β2-S1 pockets. Nevertheless, owing to the more spacious architecture of the β2-S1 pocket relative to β1-S1, MRZ exhibits modestly enhanced inhibitory potency toward the β2 subunit compared to β1.

MRZ exhibited broad-spectrum anti-tumor activity. In acute myeloid leukemia (AML) cells, it induced accumulation of p27 and p21, triggering apoptosis through FADD-mediated caspase-8 auto-processing and sequential activation of caspases-9 and -3 (Miller et al., 2011; Miller et al., 2007). This cascade was accompanied by a sustained increase in reactive oxygen species (ROS), which further amplifies the apoptotic signal (Miller et al., 2011; Miller et al., 2007). In MM, MRZ induced apoptosis in both BTZ-naïve and BTZ-refractory cells via the FADD–caspase-8 extrinsic pathway. In human MM xenograft murine models, MRZ significantly delays tumor growth and prolongs survival without causing neurological alterations after 12 weeks of treatment (Mitsiades et al., 2006; Singh et al., 2010). MRZ also displays potent pro-apoptotic effects in glioblastoma (GBM). In LN229 and U118 GBM cells, it upregulated ER-stress markers including GRP78, CHOP, and ATF4, without markedly increasing ROS, indicating that ROS are not required for MRZ-induced apoptosis (Manton et al., 2016). Furthermore, MRZ synergizes with BTZ (Mitsiades et al., 2006), cisplatin (Zhang Z. et al., 2022), pomalidomide (Das et al., 2015), and lenalidomide (Chauhan et al., 2010), demonstrating enhanced anti-tumor efficacy both *in vitro* and in xenograft models.

Beyond its anti-cancer effects, MRZ also possesses strong anti-parasitic activity. It binds the β1, β2, and β5 subunits of the Trichomonas vaginalis 20S proteasome, thereby inhibiting parasite proteolytic activity (Silhan et al., 2024). Against Leishmania amazonensis and L. infantum intracellular amastigotes, MRZ exhibited low-nanomolar activity, induced mitochondrial swelling and cytoplasmic vacuolation, and significantly reduced lesion parasite burden in BALB/c mice, with efficacy comparable to amphotericin B and without apparent hepato- or nephrotoxicity, indicating potential for anti-leishmanial drug development (Machado Patrícia de et al., 2025).

Pharmacokinetic studies reveal that MRZ is orally bioactive and capable of penetrating the blood-brain barrier (BBB) (Mitsiades et al., 2006; Di et al., 2016). In rodents, brain exposure reaches approximately 30% of plasma levels, and in non-human primates, it achieves over 30% inhibition of CT-L activity in brain tissue (Di et al., 2016). These pharmacological properties highlight MRZ’s strong potential for drug development, supporting its further optimization as an oral formulation or for the treatment of brain tumors, as will be introduced in detail in clinical studies in Section 5.

### 3.2 Peptidic epoxyketones

#### 3.2.1 Epoxomicin

Epoxomicin (Figure 4C) was isolated in 1992 from actinomycete strain Q996-17 and exhibited potent cytotoxicity against HCT-116 colon carcinoma, Moser colorectal carcinoma, and K562 myeloid leukemia cells (Hanada et al., 1992). In contrast to β-lactone inhibitors, epoxomicin utilizes its core α′,β′-epoxyketone pharmacophore to undergo nucleophilic ring opening, the compound covalently targets the N-terminal threonine of the 20S proteasome catalytic subunits, forming an irreversible, unique morpholine ring adduct with the Thr1Oγ. The absence of this nucleophilic residue in most other proteases accounts for epoxomicin’s exceptional selectivity toward the proteasome (Groll et al., 2000). Consequently, despite metabolic liabilities associated with its peptidic epoxyketone warhead, epoxomicin served as the structural prototype for the clinical proteasome inhibitor CFZ (Kim and Crews, 2013).

Among the three peptidase activities, epoxomicin most potently inhibits CT-L activity [*k*_obs_/[I] = 20,000 ± 3,000 M^-1^s^-1^ (0.037–0.1 μM)], followed by T-L [*k*_obs_/[I] = 43 ± 10 M^-1^s^-1^ (25–75 μM)] and C-L [*k*_obs_/[I] = 310 ± 70 M^-1^s^-1^ (0.25–2.5 μM)] (Meng et al., 1999; Kim et al., 1999). The hydrophobic side chain of epoxomicin (e.g., isobutyl moiety) inserts into the S1 hydrophobic pocket of the β5 subunit, inducing a conformational shift of Met45 at the pocket base and thereby expanding the hydrophobic binding interface to establish robust van der Waals interactions. Concurrently, the amide functionalities of epoxomicin engage in multiple hydrogen bonding interactions with the conserved catalytic site residues Thr21 and Gly47, further stabilizing the bound conformation of the inhibitor. In contrast, its interactions with the β1 and β2 subunits are significantly attenuated due to electrostatic repulsion from the charged S1 pockets (Arg45 in β1 and Glu53/Asp53 in β2) as well as steric constraints imposed by pocket dimensions, resulting in markedly inferior inhibitory potency relative to the β5 subunit (Supplementary Figure S1-F).

Epoxomicin is widely employed to model neurodegeneration. Systemic administration for 1–2 weeks induced progressive bradykinesia, rigidity, tremor, striatal dopamine depletion, and dopaminergic neuron loss in the substantia nigra, accompanied by α-synuclein/ubiquitin-positive inclusions resembling Lewy bodies—thereby faithfully recapitulating key pathological features of Parkinson’s disease (McNaught et al., 2004).

In addition to its neurotoxic and antitumor properties, epoxomicin exhibited pronounced anti-inflammatory activity. Daily administration of 0.58 mg/kg reduced ear swelling by 44% in a murine contact hypersensitivity model, correlating with inhibition of TNF-ɑ-induced IκBɑ degradation and suppression of NF-κB nuclear translocation and DNA binding (Meng et al., 1999). Epoxomicin also suppressed LPS/poly (I:C)-triggered activation of NF-κB and IRF3, downregulated IFN-β and IP-10, and antagonized MyD88-, IKKβ-, p65/TRIF-, TBK1-, and constitutively activated IRF3CA-mediated IRF3 signaling, indicating broad anti-inflammatory potential via disruption of the TLR-TRIF pathway (Kim et al., 2021).

Like other PIs, epoxomicin also displayed anti-parasitic activity, inhibiting Plasmodium (Czesny et al., 2009) and Babesia (AbouLaila et al., 2010) growth at nanomolar concentrations.

### 3.3 Macrolides and macrolactams

#### 3.3.1 TMC-95 family

**FIGURE 6:**
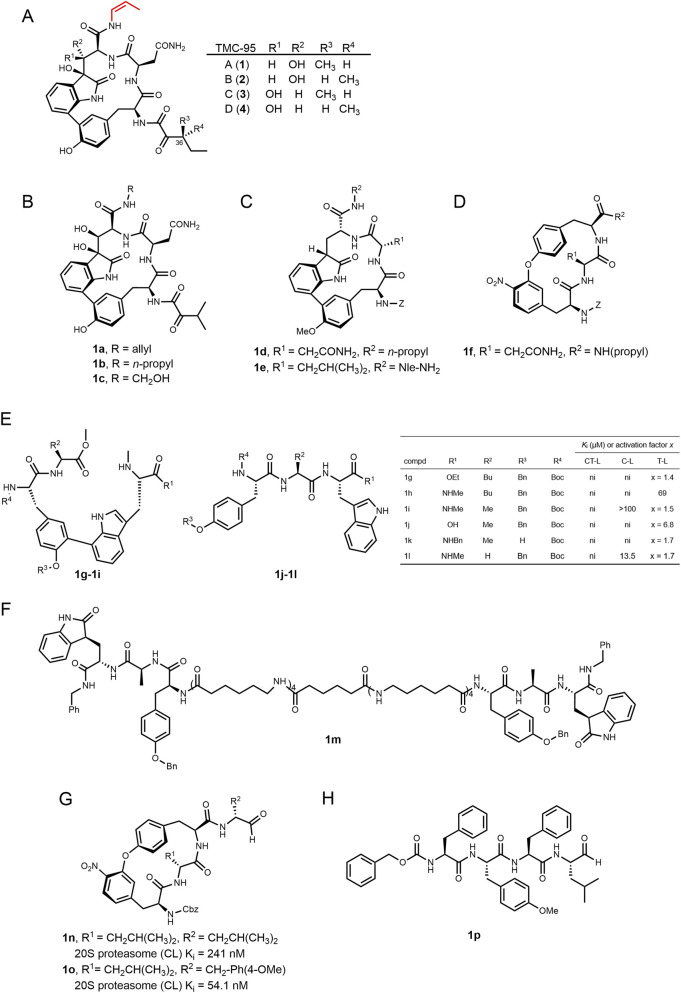
TMC-95A-D and their analogues. **(A)** TMC-95A–D. **(B–D)** Scaffold and side-chain variants 1a–1f. **(E)** Linear peptides 1g–1l with their activity data. **(F)** Bivalent analog 1m. **(G,H)** Aldehyde analogs 1n–1p. Compound warhead labeled in red.

TMC-95A, TMC-95B, TMC-95C, and TMC-95D (Figure 6) were isolated from the fermentation broth of *Apiospora montagnei Sacc*. TC 1093 (Koguchi et al., 2000) and represent a novel class of cyclic peptides composed of L-tyrosine, L-asparagine, highly oxidized L-tryptophan, (Z)-1-propenamine, and 3-methyl-2-oxopentanoic acid moieties (Yang et al., 2003). TMC-95A inhibited the 20S proteasome’s CT-L, T-L, and C-L activities with IC_50_ values of 5.4 nM, 200 nM, and 60 nM, respectively; TMC-95B displayed similar potency, whereas TMC-95C and D are 20–150-fold less active. TMC-95A exhibited remarkable selectivity, showing no inhibition against m-calpain, cathepsin L, or trypsin (Koguchi et al., 2000). Notably, this family exhibits weaker cytotoxicity than FDA-approved proteasome inhibitors. TMC-95A, the most potent member in terms of proteasome inhibition, showed cytotoxic activity against HCT-116 human colon carcinoma cells and HL-60 human promyelocytic leukemia cells, with IC_50_ values of 4.4 μM and 9.8 μM, respectively (Koguchi et al., 2000). These findings indicate that the cytotoxicity of proteasome inhibitors is not solely determined by their proteasome-inhibitory potency. Additional factors, such as permeability, also contribute.

**FIGURE 7:**
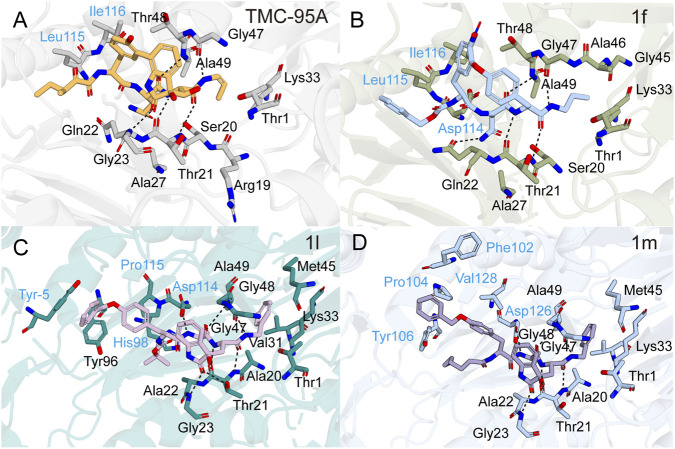
Structural interactions of TMC-95A and its analogues with the 20S proteasome. TMC-95A **(A)** (PDB: 1JD2) and its analogues 1f **(B)** (PDB: 2GPL), 1l **(C)** (PDB: 3NZW), and 1m **(D)** (PDB: 4JSQ) bound to the proteasome β2 **(A,B)** and β5 **(C,D)** subunits, respectively.

Co-crystal structures of TMC-95A bound to yeast 20S proteasome reveal that the (Z)-propenyl chain occupies the S1 pocket, while the asparagine side chain extends into S3, forming an antiparallel β-sheet stabilized by multiple hydrogen bonds in a non-covalent mode (Figure 7A). TMC-95A achieves 100% occupancy at the binding sites of the proteasomal β1, β2, and β5 subunits, as confirmed by temperature factor refinement. Notably, β2 is the subunit most weakly inhibited by TMC-95A, with the selectivity profile being β5 > β1 > β2. However, high-resolution electron density of TMC-95A is exclusively observable at the β2 subunit (PDB: 1JD2), thus only the binding mode between TMC-95A and the β2 subunit can be clearly defined (Groll et al., 2001). Because of its non-covalent yet highly selective binding profile, TMC-95A serves as an important lead compound for proteasome inhibitor development. Although total synthesis has been achieved (Lin and Danishefsky, 2002), the molecule’s complex macrocyclic framework, multiple sensitive substituents, and low overall yield make analog development essential. Structure-activity relationship (SAR) analyses demonstrated that the C36 stereocenter had little effect on potency, and substituting the sec-butyl group with isobutyl or isopropyl did not alter *K*_iapp_, eliminating the need for strict stereochemical control in later optimizations. In contrast, replacing the metabolically labile propenyl group with n-propyl or alcohol side chains (**1a**–**c**) reduced potency: n-propyl substitution decreases CT-L activity by ∼15-fold and C-L by ∼4-fold, while alcohol derivatives exhibited only micromolar activity, underscoring the critical role of the olefin moiety (Yang et al., 2003).

Guided by the 20S-TMC-95A co-crystal structure, researchers designed a minimal core scaffold by replacing the N-terminal alkyl ketoamide with benzyl urethane, removing the two tryptophan hydroxyls, and methylating the tyrosine phenol, thereby simplifying synthesis. Derivatives **1d** and **1e**, in which (Z)-propenyl is replaced by n-propyl or Nle-NH_2_ and asparagine by leucine, showed 3–2000-fold reductions in potency, mainly due to disruption of key hydrogen bonds and steric clashes with Met45 in the S1 pocket (Kaiser et al., 2004a). Replacement of the phenol-oxindole biaryl with an endocyclic biaryl (**1f**) abolished the hydrogen bond bridged by the CO of the oxindole to the NH of Gly23, destabilizing the backbone conformation (Figure 7B). Nevertheless, **1f** retained activity comparable to **1d**, indicating that macrocyclic remodeling is feasible (Groll et al., 2006a; Kaiser et al., 2004b).

To simplify synthesis, linear analogs of TMC-95A (**1g-1l**) were prepared by cleaving the Tyr/oxidized Trp or oxidized Trp/Asn junctions, leading to marked activity attenuation (Basse et al., 2007). In compound **1l**, the phenyl ring deeply inserts into the hydrophobic β5-S1 pocket, forming van der Waals interactions with Met45, Val31, and Ala49 of β5; the oxindole group establishes specific hydrogen bonds with the backbone NH group of Gly23 and side-chain OH group of Thr21 in β5; and the Tyr-Asn-oxidized Trp tripeptide core adopts an antiparallel β-sheet conformation with the β5 main chain (Figure 7C). Despite a similar binding mode to TMC-95A, this linear decarboxylated analog has high backbone flexibility that necessitates conformational rearrangement for pocket binding, and lacks a side chain to penetrate the S3 pocket as the asparagine of TMC-95A does, leading to a significant reduction in overall binding affinity (Groll et al., 2010).

Linearization (**1g**–**l**) drastically reduces activity. However, dimeric linear mimics (**1m**) connected by amino-hexanoic/adipic acid spacers and re-introducing the oxindole restored low-nanomolar potency comparable to TMC-95A (Groll et al., 2010; Desvergne et al., 2013). Although structural analysis reveals that dimeric monomers retain interactions with β subunits through hydrophobic contacts at S1 and S4 pockets and antiparallel β-sheet formation (Figure 7D), Lineweaver-Burk plot analysis indicates that, unlike the mixed-type or non-competitive inhibition exhibited by monomers, dimeric inhibitors display competitive inhibition. This suggests that bivalent binding more effectively occupies the substrate-binding pocket, obstructing substrate-enzyme interaction and thereby markedly enhancing inhibitory activity (Desvergne et al., 2013).

Furthermore, conversion of the P1 amide into a peptidic aldehyde (**1n**–**p**) substantially enhanced 20S inhibition. Notably, linear aldehyde **1p** achieved CT-L potency similar to CFZ but exhibited increased off-target inhibition of cathepsin B and m-calpain, suggesting that the macrocyclic architecture of TMC-95A is essential for proteasome selectivity (Wilson et al., 2016).

#### 3.3.2 Syringolin A/B

**FIGURE 8:**
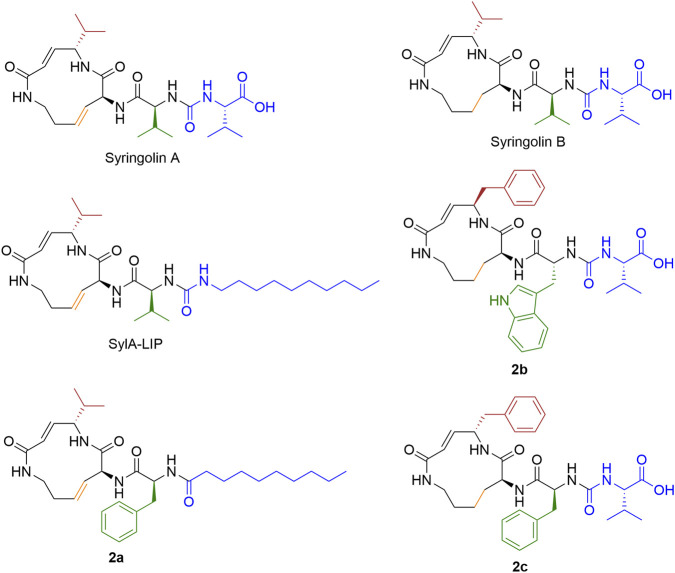
Syringolin A/B and their analogues. Structures of syringolin A/B and their analogues were demonstrated. The regions of structural modification and optimization in each compound are highlighted in different colors. Compound warhead labeled in red.

Syringolin A (SylA) and its analogues SylB-F (Figure 8) were identified in rice (*Oryza sativa* L.) as defense metabolites activating innate immunity against the blast fungus *Pyricularia oryzae* (Wäspi et al., 1998; Wäspi et al., 1999). SylA selectively inhibits the yeast 20S proteasome, showing the highest potency toward the CT-L activity [*k*_assoc_ = 863 ± 106 M^-1^s^-1^ (100–200 nM)], followed by T-L [*k*_assoc_ = 94 ± 12 M^-1^s^-1^ (150–600 μM)] and C-L activities [*k*_assoc_ = 6 ± 0.3 M^-1^s^-1^ (20–40 μM)], while exhibiting no inhibition of papain or trypsin, confirming its proteasome specificity (Groll et al., 2008b; Clerc et al., 2009a). The co-crystal structure of the 20S proteasome with SylA revealed Michael 1,4-addition of Thr1Oγ to the SylA C4 α,β-unsaturated carbonyl double bond, forming a covalent ether. Additional hydrogen bonding between Gly47NH and the carbonyl group, together with an antiparallel β-sheet interaction, stabilized the adduct and underpins irreversible inhibition. The subunit selectivity of SylA arises from the structural complementarity between its rigid 12-membered macrocycle and the respective S1 pockets of different proteasomal subunits. The β5, featuring a hydrophobic and spacious S1 pocket, can stably accommodate the 12-membered macrocycle and enable the dipeptide moiety to maintain a planar conformation for β-sheet formation. The β2-S1 pocket, though spacious, exerts electrostatic repulsion on the 12-membered macrocycle, thus impeding the formation of a stable β-sheet. The β1-S1 pocket, however, exerts both electrostatic repulsion and steric hindrance, causing conformational change of the dipeptide moiety and thus preventing the assembly of a stable β-sheet (Supplementary Figure S1G-I). Consequently, the inhibitory potency of SylA toward the β5 subunit is remarkably superior to that toward the β2 subunit, with the weakest efficacy against the β1 subunit (Clerc et al., 2009a).

SylB, which lacks the reactive double bond, form a slowly reversible ether linkage but displays weaker inhibition, while preserving the subunit selectivity profile of SylA [CT-L: *k*_assoc_ = 122 ± 31.7 M^-1^s^-1^ (1.5–3.1 μM), T-L: *k*_assoc_ = 4.6 ± 0.7 M^-1^s^-1^ (50–100 μM), C-L: not active at a concentration up to 150 μM] (Clerc et al., 2009b). Loss of the double bond ablates the macrocycle’s ring strain and rigidity, causing its displacement from the active site; the concomitant conformational change of the dipeptide moiety precludes optimal β-sheet backbone alignment, disrupts the antiparallel β-sheet interaction, and thus fails to afford a stable residency for covalent conjugation (Supplementary Figure S1H, I) (Clerc et al., 2009b).

Current optimization efforts focus on the N-acyl chain and macrolactam core. As SylA and its methyl ester are equipotent, the free carboxylate group is dispensable (Clerc et al., 2009b). Hybridization of the SylA macrocycle with the GlbA side chain (SylA-GlbA) produced a dual β2/β5 inhibitor, suggesting that the syrbactin macrocycle modulates subsite selectivity (Clerc et al., 2011). First-generation SylA-LIP, in which the 3-methylbutanoyl group was replaced with a lipophilic alkyl chain, enhanced β2/β5 inhibition by approximately 100-fold (Clerc et al., 2009b). Structure-guided expansion of the S3 hydrophobic pocket afforded 18-N-benzyl analog **2a** (decyl side chain), which showed a 15-fold improvement in CT-L inhibition and lipophilicity-driven cytotoxicity equipotent to BTZ in HCT116, A431, and RPMI8226 cells. Notably, **2a** retained activity against BTZ-resistant KMS-11/BTZ and OPM-2/BTZ cells and, like BTZ, induced accumulation of phosphorylated and polyubiquitinated IκBɑ. It was also well tolerated in mice, highlighting its therapeutic promise (Chiba et al., 2014; Yoshida et al., 2016).

Substrate-mimetic optimization of SylB by replacing the isopropyl group at C3 of the lactam with an indole-2-methyl moiety generated derivative **2b**, which exhibited a 5.5-fold increase in the second-order inhibition rate constant and an 11.5-fold enhancement in cytotoxicity against leukemia cell lines relative to SylB, supporting the effectiveness of a substrate-guided design approach (Totaro et al., 2015). Systematic C3 substitutions revealed that hydrophobic substituents abolished β2 activity, basic groups decreased β5, but enhanced β2 inhibition, and acidic groups reduced both, while polar substituents showed variable effects. Notably, cellular IC_50_ values do not always correlate with enzymatic inhibition, likely due to differences in membrane permeability—for example, benzyl analog **2c** exhibited the lowest IC_50_ in prostate cancer LNCaP and DU145 cells (Tatsumi et al., 2024).

Collectively, these findings indicate that the macrolactam side chain dictates peptidase selectivity and cytotoxicity, whereas N-acyl lipophilicity enhances cellular permeability. Lead analogs **2a** and **2c** achieve low-nanomolar cellular potency. However, detailed mechanistic, *in vivo* efficacy, and toxicology evaluations remain to be completed.

### 3.4 Other types of natural product-derived proteasome inhibitors

In addition to the systemically studied inhibitors described above, a growing number of natural products exert biological effects at least in part by inhibiting the proteasome.

#### 3.4.1 Shikonin

**FIGURE 9:**
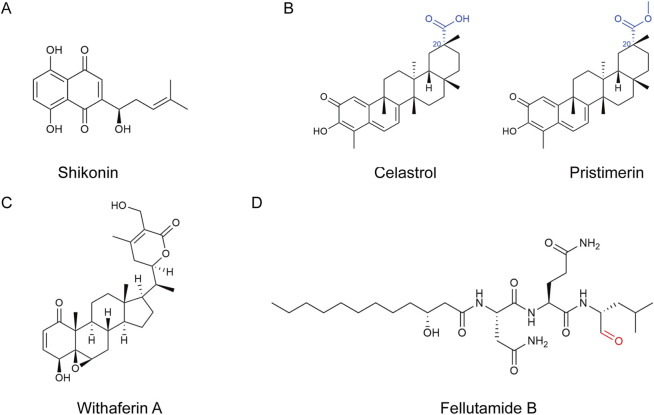
Structures of other types of natural product-derived proteasome inhibitors. **(A)** Structure of shikonin, a naphthoquinone isolated from the roots of *Lithospermum erythrorhizon* Siebold and Zucc. and *Arnebia euchroma* (Royle) I.M.Johnst., was demonstrated. **(B)** Structures of celastrol and pristimerin (Celastrol methyl ester) were demonstrated. The differing substituents between the two compounds are highlighted in blue. **(C)** Structure of withaferin A, a steroidal lactone from *Withania somnifera,* was demonstrated. **(D)** Structure of fellutamide B, isolated from *Penicillium fellutanum,* was demonstrated. Fellutamide B warhead labeled in red.

Shikonin (Figure 9A), a naphthoquinone isolated from the roots of *Lithospermum erythrorhizon* Siebold and Zucc. and *Arnebia euchroma* (Royle) I.M.Johnst., inhibited rabbit 20S CT-L activity (Yang et al., 2009) and displayed cytotoxicity against PC-3 prostate cancer cells, P388 leukemia cells, H22 hepatoma cells, MDA-MB-231 breast cancer cells, and several MM cell lines (Yang et al., 2009; Wang A. et al., 2021; Wada et al., 2015; Li et al., 2022). Its anti-tumor mechanisms involve accumulation of pro-apoptotic proteins (such as IκBɑ, Bax, and p27), ubiquitin-dependent degradation of cIAP1/2, disruption of the cIAP-RIP1 interaction, degradation of RIP1, upregulation of TNF-α, induction of ER stress (XBP-1 splicing), and increased expression of HSP70/72 (Yang et al., 2009; Wang A. et al., 2021). In rat primary macrophages, shikonin blocked p65-NF-κB nuclear translocation and promoted the accumulation of ubiquitinated proteins, thereby inducing macrophage apoptosis and exhibiting anti-inflammatory activity (Lu et al., 2011).

#### 3.4.2 Celastrol

Celastrol and pristimerin (Celastrol methyl ester) are quinone-methide triterpenoids derived from *Tripterygium wilfordii* Hook.f., species of the Celastraceae families (Dai et al., 2023). These compounds differ only at the C20 position, where celastrol contains a carboxyl group and pristimerin a methyl ester (Figure 9B), yet they share identical mechanisms of action: selective inhibition of 20S proteasome CT-L activity, blockade of the ubiquitin–proteasome pathway, accumulation of IκBɑ, Bax, and p27, suppression of NF-κB signaling, and downregulation of the androgen receptor (AR) (Yang et al., 2008; Yang et al., 2006). Celastrol exhibited low micromolar to nanomolar IC_50_ against prostate cancer cell lines (Dai et al., 2010) and helped to enhance the efficacy of paclitaxel in a liposomal co-delivery system (Xiao et al., 2022), whereas pristimerin showed nanomolar potency in MM cells (Tiedemann et al., 2009), highlighting their promise as proteasome-targeted therapeutic leads.

#### 3.4.3 Withaferin A

Withaferin A (WA, Figure 9C), a steroidal lactone from *Withania somnifera* (L.) Dunal, inhibited T-L activity, blocked the ubiquitin-proteasome pathway, and caused accumulation of ubiquitinated proteins and pro-apoptotic factors (Yang et al., 2007; Yang et al., 2012; Xing et al., 2023). Proteasome inhibition by WA induced the expression of ER-resident chaperones (BiP, GRP94) and heat-shock proteins (HSP70, HSP30), with BiP perinuclear puncta and HSP30 granular deposition, indicating concurrent ER and cytosolic proteotoxic stress (Khan et al., 2012). Additionally, WA inhibited the ubiquitin-dependent degradation of IDH1, thereby stabilizing the enzyme, increasing α-ketoglutarate (α-KG) levels, enhancing prolyl-hydroxylase (PHD) activity, and leading to inactivating the HIF-1α signaling (Xu et al., 2019).

#### 3.4.4 Fellutamide B

Fellutamide B (Figure 9D), isolated from *Penicillium fellutanum*, formed a hemi-acetal adduct between its aldehyde group and the Thr1γ-O of the yeast (PDB: 3D29) (Hines et al., 2008) and *Mycobacterium tuberculosis* (PDB: 2FHG) (Lin et al., 2010) 20S proteasomes. As a peptidyl aldehyde inhibitor, fellutamide B forms a reversible hemiacetal linkage with the Thr1Oγ residue via its reactive aldehyde moiety, while its Gln, Leu, and Asn residues engage in hydrogen bonding interactions with the β5 subunit. Notably, fellutamide B achieves selectivity toward the catalytic subunit through an unprecedented mechanism involving interactions with a neighboring non-catalytic subunit: its aliphatic tail establishes robust van der Waals interactions within a hydrophobic groove defined by Pro95, Tyr96, Pro115, and Val116 of the β6 subunit, thereby substantially enhancing its binding affinity for β5 (IC_50_ = 9.4 ± 2.5 nM). In contrast, upon binding to the β1 and β2 subunits, the aliphatic tail adopts a markedly distinct conformation, relying solely on antiparallel β-sheet formation mediated by the peptidic backbone for subunit stabilization, resulting in significantly attenuated inhibitory potency (IC_50_ = 2.0 ± 0.4 μM and 1.2 ± 0.8 μM, respectively) (Supplementary Figure S1J-L). Proteasome-specific inhibition, rather than membrane depolarization or cytoskeletal disruption, uniquely induced nerve growth factor (NGF) expression and stimulated NGF mRNA transcription through two distinct promoters, although the detailed molecular mechanism remains to be elucidated (Hines et al., 2008).

#### 3.4.5 Other less explored natural products with proteasome-inhibitory activity

In addition to the well-characterized natural product-derived PIs discussed above, numerous other natural products have also been reported to possess proteasome-inhibitory activity. Some of these compounds have been further investigated for their effects on downstream signaling pathways, such as NF-κB suppression, ER stress induction, ROS generation, and apoptosis activation (Table 3).

**TABLE 3:** Less explored natural products inhibiting proteasome activity and their bioactivities.

Name	Source	Proteasome inhibition	Biological effects
Apigenin	Fruits, vegetables	CT-L	IκBα accumulation, Bax upregulation (Chen et al., 2007)
Physalin B	*Physalis angulata* L	CT-L and C-L	NF-κB suppression, NOXA induction, apoptosis (Vandenberghe et al., 2008)
Sanggenon C	*Morus cathayana* Hemsl	CT-L	Apoptosis, cell-cycle arrest (Huang et al., 2011)
Radix Tetrastigma Hemsleyani Flavone	*Tetrastigma hemsleyanum* Diels and Gilg	T-L	↑Ubiquitinated proteins: Bax; ↓Bcl-2, USP14, UCHL5, POH1 (Zhong et al., 2019)
Polyphyllin I	*Paris polyphylla* Sm	Selective CT-L	NF-κB blockade, ER stress/UPR, cell-cycle inhibition, apoptosi (Chang et al., 2015)
Methylferulate	*Tamarix aucheriana* Decne	Selective CT-L and C-L	Apoptosis, ROS, NF-κB DNA-binding inhibition, Cdk1/2 modulation (Abaza et al., 2016)
Marchantin M	*Asterella angusta* (Steph.) Pandé, K.P.Srivast. and Sultan Khan (Qu et al., 2007)	Selective CT-L and C-L	PERK/eIF2α-mediated ER stress, autophagy, cell death (Jiang et al., 2013)
Fangchinoline	*Stephania tetrandra* S.Moore	C-L	Accumulation of Ub-IκBα, Ub-p27, Ub-Bax; cycle arrest, apoptosis; prostate xenograft suppression (Li et al., 2015a)
Tannic acid	Widespread trees and plants (Hosseini et al., 2025)	CT-L	p27^Kip1^ and Bax accumulation, G1 arrest, apoptosis (Nam et al., 2001)
Baceridin	*Epiphytic Bacillus* Strain	T-L, CT-L, C-L	p53-independent apoptosis, cycle arrest (Niggemann et al., 2014)
Comaparvin	*Comanthus* sp.	CT-L	NF-κB inhibition (activation, phosphorylation, nuclear translocation, DNA binding, target genes); TNF-α-induced IκBα phosphorylation/degradation and IKKβ suppression (Chovolou et al., 2011)
Isoginkgetin	*Ginkgo biloba* L. (Chen et al., 2025)	CT-L, T-L, C-L	NF-κB blockade, ER stress and autophagy, lysosomal disruption, MM apoptosis (Tsalikis et al., 2019)
Plumbagin	*Plumbago indica* L	CT-L	ER stress, thiol imbalance, ↓Δψm and ATP, paraptosis (Binoy et al., 2019)
Palau’amine	*Stylotella agminata* (Kinnel et al., 1993)	CT-L and C-L, irreversible 20S binding	*In-vivo* accumulation of ubiquitinated proteins (Lansdell et al., 2012)

Several natural products have been only preliminarily studied, with available data limited to their proteasome-inhibitory and cytotoxic activities (Table 4). Although most of these compounds display only micromolar potency, their diverse chemical scaffolds and multifaceted biological mechanisms provide valuable templates and strategic directions for the discovery and optimization of next-generation PIs.

**TABLE 4:** Survey of less-explored natural product proteasome inhibitors.

Investigation	Names
Proteasome only	Variabines B (Sakai et al., 2014), pyrrolizilactone (Futamura et al., 2013), cystargolides (Gill et al., 2015), mellains A and B (Nishimura et al., 2022), kadsurenin F (Evangelakou et al., 2025), tomatine and tomatidine (da Silva et al., 2017)
Proteasome and cytotoxicity	Carmaphycins A and B (Pereira et al., 2012), tetradehydrohalicyclamine B (Kato et al., 2019), piperlongumine (Bosc et al., 2018), macyranone A (Keller et al., 2015), ajoene (Xu et al., 2004)

Collectively, β-lactones, peptidic epoxyketones, macrolides, macrolactams, and other representative natural product-derived proteasome inhibitors exhibit distinct structural features and biological properties. A systematic comparison of their core characteristics is provided (Table 5).

**TABLE 5:** Core characteristics of proteasome inhibitors.

Classes of natural products	Name	Binding mode	Key strength	Primary translational barrier
β-Lactones	Lactacystin	Slowly reversible covalent ester linkage (Moore et al., 2008)	Peptide-free structure, avoids broad off-target effects of peptide inhibitors (Fenteany et al., 1995)	Unstable active form of lactacystin (omuralide) (Ōmura and Crump, 2019), potential neurotoxicity (McNaught et al., 2002; Savolainen et al., 2017)
	Marizomib	Irreversible covalent ester linkage (Groll et al., 2006b)	Oral bioavailability (Chauhan et al., 2010) and BBB penetration (Di et al., 2016)	Central neurotoxicity, limited efficacy in solid tumors (Stupp et al., 2005), (NCT: 03345095)
Peptidic Epoxyketones	Epoxomicin	Irreversible covalent morpholino adduct (Groll et al., 2000)	Highly selective covalent binding to proteasome catalytic subunits N-terminal Thr1 (Groll et al., 2000)	Poor drug-like properties (Kim and Crews, 2013)
Macrolides and Macrolactams	TMC-95A–D	Non-covalent hydrogen-bonding networks (Groll et al., 2001)	Selective non-covalent natural product proteasome inhibitor (Koguchi et al., 2000)	Difficult synthesis, low yield (Lin and Danishefsky, 2002)
	Syringolin A/B	Irreversible/Slowly reversible ether linkage (Moore et al., 2008)	12-membered macrocycle, conformational restriction for selective binding (Clerc et al., 2009b)	Difficult synthesis, poor membrane permeability (Clerc et al., 2009b)
Other types	Fellutamide B	Reversible covalen hemiacetal linkage (Hines et al., 2008)	Distinctive β5-targeting selectivity through β6 subunit interaction (Hines et al., 2008)	Potential neurotoxicity (Hines et al., 2008)

## 4 Natural products synergizing with proteasome inhibitors via multiple mechanisms

**FIGURE 10:**
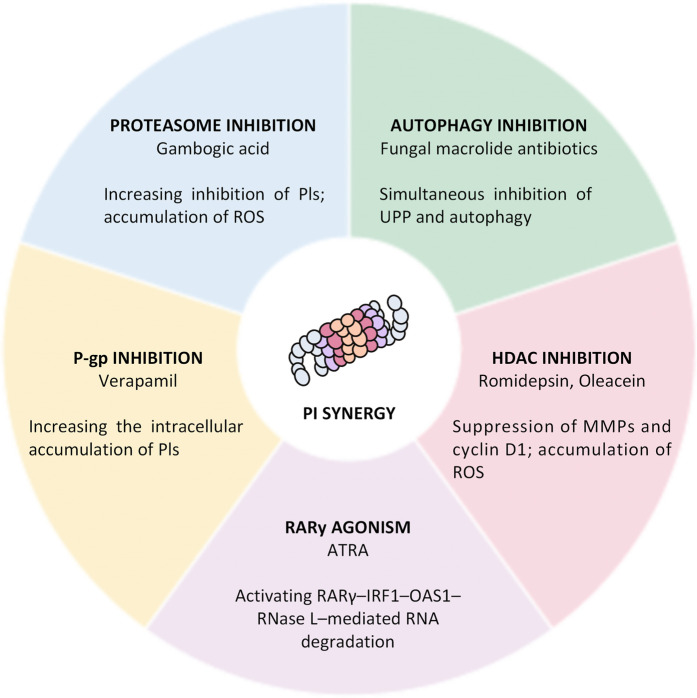
Mechanisms underlying the synergy of natural products with proteasome inhibitors. Natural products enhance the effect of PIs through different Mechanisms, including proteasome inhibition, autophagy inhibition, HDAC inhibition, RARγ agonism, and P-glycoprotein (P-gp) inhibition.

Natural products not only act as direct PIs but also sensitize cells and murine models to proteasome inhibition by regulating multiple cellular signaling pathways. Representative examples of these synergistic interactions are summarized below (Figure 10).

### 4.1 Proteasome inhibition

Distinct PIs exhibit differential affinities for individual catalytic subunits, diverse pharmacokinetic profiles, and variable specificity toward efflux pumps. Consequently, combining two mechanistically distinct inhibitors can produce synergistic effects through complementary actions.

**FIGURE 11:**
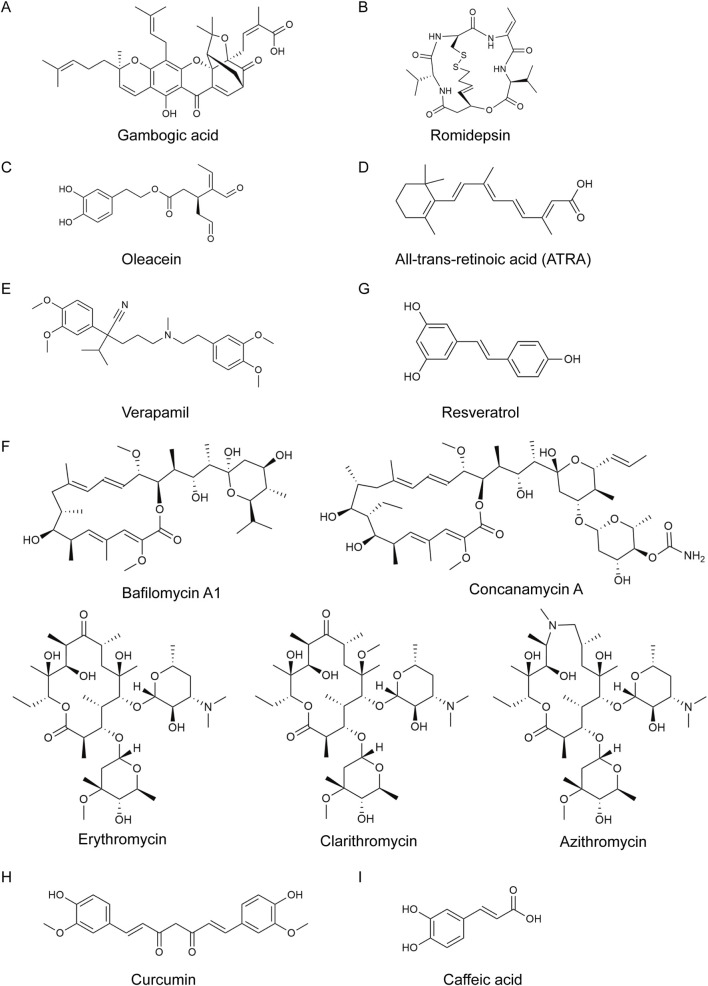
Structures of natural products synergizing with proteasome inhibitors. **(A)** Structure of gambogic acid, isolated from *Garcinia hanburyi* Hook.f., was demonstrated. **(B)** Structure of romidepsin, isolated from *Chromobacterium violaceum*, was demonstrated. **(C)** Structure of oleacein, a semisynthetic secoiridoid derived from the olive metabolite oleuropein, was demonstrated. **(D)** Structure of ATRA, an endogenous metabolite of vitamin A, was demonstrated. **(E)** Structure of verapamil, a papaverine derivative (isolated from *Papaver somniferum* L.), was demonstrated. **(F)** Structures of fungal macrolide antibiotics bafilomycin A1, concanamycin A, erythromycin, clarithromycin, and azithromycin were demonstrated. **(G)** Structure of resveratrol, a polyphenolic stilbene found in grapes (*Vitis vinifera* L.) and red wine, was demonstrated. **(H)** Structure of curcumin, isolated from *Curcuma longa* L., was demonstrated. **(I)** Structure of caffeic acid, a constituent of propolis extracts, was demonstrated.

Gambogic acid (GA, Figure 11A), the major bioactive component of *Garcinia hanburyi* Hook.f., undergoes CYP2E1-mediated biotransformation to the reactive metabolite MT1, which inhibits the proteasomal β5 chymotryptic subunit via interaction with Thr1 of β5 (Li et al., 2013). In human HepG2 and murine H22 tumor cell lines, GA synergized with BTZ to induce a rapid accumulation of intracellular reactive oxygen species (ROS), activating the PERK–eIF2α–ATF4 axis and upregulating CHOP along with proapoptotic genes such as BIM and BAX. However, in HepG2 xenografts and H22 allograft models, the antitumor efficacy of the GA/BTZ combination was not consistent with the *in vitro* findings, underscoring the need for further pharmacokinetic optimization (Liu et al., 2014).

### 4.2 HDAC inhibition

Although the precise mechanisms underlying the synergy between histone deacetylase (HDAC) inhibitors and PIs remain incompletely defined, current evidence suggests that HDAC inhibitors potentiate PI-induced proteotoxic stress, while PIs reciprocally suppress class I HDAC expression (McConkey, 2010). Nevertheless, further studies are required to fully elucidate the molecular basis of this bidirectional interaction.

Romidepsin (Figure 11B), a natural product isolated from *Chromobacterium violaceum*, is a potent class I/II HDAC inhibitor (VanderMolen et al., 2011). Combination treatment with romidepsin and BTZ markedly reduced cell viability of A549 cells, increasing the sub-G_0_/G_1_ fraction. Upregulation of p21 and p53 indicated that activation of the p53/p21 axis mediated G1 arrest and amplified apoptotic signaling. Moreover, combination of romidepsin and BTZ suppressed MMP-2 and MMP-9 expression, suggesting attenuation of extracellular matrix degradation, consequently, diminished invasive potential. Global hyperacetylation of histones H3 and H4 not only reflects effective HDAC inhibition but may also activate chromatin-remodeling-dependent accessory pathways that converge on PI-induced apoptotic death (Karthik et al., 2014). In mantle cell lymphoma, romidepsin-driven histone H3 acetylation repressed cyclin D1 transcription, whereas BTZ stabilized p27 and p21, culminating in synergistic accumulation of CDK inhibitors and profound anti-proliferative effects (Paoluzzi et al., 2010).

Oleacein (Figure 11C), a semisynthetic secoiridoid derived from the olive metabolite oleuropein (isolated from *Olea europaea L.*) (Filardo et al., 2024), also exhibited HDAC inhibitory activity and induced dose-dependent cytotoxicity in MM cells while sparing peripheral blood mononuclear cells from healthy donors. Combination index (CI) analyses revealed synergy between oleacein and CFZ, but antagonism with BTZ (Juli et al., 2019). This discrepancy may arise from oleacein’s antioxidant properties (Parzonko et al., 2013): CFZ elicited a greater ROS surge than BTZ (Fink et al., 2016), and moderate ROS levels remained cytotoxic when oleacein was co-administered with CFZ, whereas excessive ROS quenching in the BTZ/oleacein regimen dampened the lethal signal. Additionally, BTZ can activate autophagy and redirect ubiquitinated substrates (including IκBα) toward autophagosomes, thereby potentiating canonical NF-κB signaling pathway (Pakjoo et al., 2024). Moreover, oleacein independently suppressed NF-κB activation (Cirmi et al., 2022), potentially provoking a compensatory cytoprotective autophagy that attenuated BTZ-driven apoptosis.

### 4.3 RARγ agonism

All-trans-retinoic acid (ATRA) (Figure 11D), an endogenous metabolite of vitamin A, exerted negligible toxicity in MM cells yet markedly augmented CFZ-induced apoptosis (Wang et al., 2022). Multiple myeloma cell lines abundantly express RARγ, RXRβ, and RXRγ. Although ATRA functions as a pan-RAR agonist, its synergistic effect with CFZ was primarily mediated through RARγ activation. Gene-set enrichment analysis revealed significant activation of the IFN-β pathway, with concomitant induction of OAS1 and IRF1. IRF1, a pivotal transcriptional effector downstream of IFN-β, was indispensable for combination efficacy. The knockdown of IRF1 attenuated ATRA/CFZ-induced apoptosis from ∼65% to ∼25%. Moreover, IRF1 binding to the OAS1 promoter drived OAS1 transcription. Upon sensing CFZ-induced double-stranded RNA, OAS1 synthesized 2′-5′-oligoadenylates that activate RNase L, leading to global RNA degradation. Collectively, the RARγ–IRF1–OAS1–RNase L pathway is central to ATRA-mediated sensitization to CFZ (Wang et al., 2022).

### 4.4 P-glycoprotein inhibition

Verapamil (Figure 11E), a papaverine derivative (isolated from *Papaver somniferum* L.) and potent P-gp inhibitor, blocks the drug-efflux function of P-gp on tumor-cell membrane, thereby markedly increasing the intracellular accumulation of PIs such as BTZ and CFZ in triple-negative breast cancer MDA-MB-231 cells. This accumulation amplified the inhibitors’ suppression of 20S proteasomal chymotrypsin-like activity, further increased the levels of pro-apoptotic substrates (ubiquitinated proteins, IκBα, and p27kip1), triggered PARP cleavage and caspase-3/7 activation, and produced synergistically enhanced cytotoxicity and apoptosis—demonstrating a cooperative lethality that arises from the simultaneous inhibition of P-gp and the proteasome (Deshmukh et al., 2017).

### 4.5 Autophagy modulation

The fungal macrolide antibiotics bafilomycin A1, concanamycin A (CAM), azithromycin, erythromycin, and clarithromycin (Figure 11F) function as autophagy inhibitors. Given that BTZ can induce autophagy, combining macrolide antibiotics with BTZ resulted in elevated LC3B-I/II ratios and p62 accumulation, indicative of autophagic flux blockade (Moriya et al., 2013). Co-treatment with CAM and BTZ enhances cytotoxicity and apoptosis, increases ubiquitinated proteins, and upregulates CHOP together with its transcriptional targets (BIM, BAX, DR5, TRB3). These data indicate that the synergistic mechanism of the CAM/BTZ combination arises from the simultaneous inhibition of the two major protein degradation systems, resulting in heightened ER stress and subsequent CHOP-dependent apoptotic signaling (Moriya et al., 2013).

Conversely, autophagy inducers demonstrate different effects in combination with PIs at different doses. Resveratrol (Figure 11G), a polyphenolic stilbene found in grapes (*Vitis vinifera* L.) and red wine, exhibited biphasic effects: at low concentrations, it protected against MG132-, PSI-, lactacystin-, and epoxomicin-induced cytotoxicity (Niu et al., 2011), whereas at high concentrations, it synergized with CFZ to promote apoptosis (Li Q. et al., 2018). Cell-cycle analysis further revealed that MG132/resveratrol co-treatment induced G1-phase arrest accompanied by a reduction of S-phase cells (Niu et al., 2011), whereas CFZ/resveratrol increased the percentage of cells in G2/M phase (Li Q. et al., 2018), suggesting that the specific cell-cycle context may determine the ultimate cytotoxic outcome.

### 4.6 Other mechanisms

Despite the synergistic mechanisms summarized above, numerous natural products interact with PIs through mechanisms that remain incompletely understood, offering an expansive and highly promising reservoir for future mechanistic investigation and therapeutic refinement.

Curcumin (Figure 11H), isolated from *Curcuma longa* L., has been reported to exhibit multiple biological activities, including anti-inflammatory, anti-tumor, neuroprotective, and hepatoprotective effects (Zhao et al., 2023; Wei et al., 2024; Zhang Y. et al., 2022; Cao et al., 2025). Interestingly, researchers also found that curcumin cooperated with CFZ to induce G1 arrest and augment apoptosis (Allegra et al., 2018). Compared with the curcumin/BTZ combination, its water-soluble amino-acid-conjugated analogue #12 in combination with BTZ achieves complete 26S proteasome inhibition and elicits markedly stronger anti-proliferative and proapoptotic effects (Mujtaba et al., 2012). The natural anthraquinone derivative emodin combined with CFZ (Hsu et al., 2022), and indole-3-carbinol from cruciferous vegetables combined with BTZ (Taylor-Harding et al., 2012), markedly reduced cell viability, induced cell-cycle arrest, and elevated expression of apoptosis-related proteins. The brown seaweed polysaccharide fucoidan also exhibited synergistic effects with BTZ in breast cancer cell lines MCF-7 and MDA-MB-231 (Rani et al., 2025).

Interestingly, although caffeic acid (CFA, Figure 11I), a constituent of propolis extracts, alone failed to synergize with BTZ (Rani et al., 2025), a HP-PCL (N-2-hydroxypropyl methacrylamide–polycaprolactone) polymeric micellar (PMCs) system for the simultaneous delivery of BTZ and CFA converted *in vitro* antagonism to *in vivo* synergy. This reversal may arise from pH-dependent boronate chemistry: at physiological pH (7.4), the 1,2-diol moiety of CFA formed a reversible boronic ester with the boronic acid of BTZ, whereas in the acidic tumor microenvironment (≈pH 5.6), hydrolysis of the ester reactivated BTZ, allowing the co-release of both the proteasome inhibitor and the P-gp-modulating CFA (Rani et al., 2023). These findings underscore how rational drug-delivery systems can unlock latent cooperative interactions and broaden the therapeutic landscape for natural product/proteasome inhibitor combinations.

## 5 Clinical trials of natural-product proteasome inhibitor—Marizomib

MRZ, the only natural product-derived PI to have advanced to clinical trials, has received orphan-drug designation for malignant glioma by the FDA and for MM by the European Medicines Agency. Over the past decades, MRZ has been evaluated both as a monotherapy and in combination regimens in clinical trials.

Clinical trials have consistently shown that MRZ exhibits modest single-agent activity in RRMM. In a phase I clinical trial (NCT00461045) involving 27 RRMM patients who received doses ranging from 0.025 to 0.7 mg/m^2^, eight patients maintained stable disease for 6–15 months without significant toxicity (Richardson et al., 2009). Notably, no myelosuppression, peripheral neuropathy, or thrombosis was observed, confirming a favourable tolerability profile (Richardson et al., 2009). A subsequent phase II trial (NCT00461045) in 15 patients reported an objective response rate of ∼27% and a disease control rate (≥ stable disease) of 74%; the most frequent severe adverse event was fatigue (Clinicaltrials.Gov, 2007; Richardson et al., 2009). Another phase I study (NCT00629473) demonstrated objective responses in four of 27 patients (1 very good partial response and three partial responses) with a median response duration of 27 weeks. In the recommended-dose cohort (n = 10), the median PFS was 20.4 weeks, and treatment-related toxicities were primarily grade 1–2 fatigue, nausea, diarrhoea, and injection-site pain, with no drug-related deaths (Harrison et al., 2016). Collectively, these studies confirm that MRZ has limited monotherapy efficacy in RRMM but an excellent safety and neurotoxicity profile, suggesting potential value in combination strategies.

In a subsequent phase I trial (NCT02103335), 38 RRMM patients received MRZ in combination with pomalidomide and low-dose dexamethasone (Spencer et al., 2016). Despite a median of four prior treatment lines and dual resistance to lenalidomide and BTZ in 53% of participants, the regimen achieved an ORR of 53% and a clinical benefit rate of 64%, with retained efficacy in triple-refractory and high-risk cytogenetic subgroups. Neutropenia was the most common adverse event, but the addition of MRZ did not increase the frequency or severity of toxicities associated with the backbone regimen. No dose-limiting toxicities (DLTs) were attributed to MRZ, confirming the feasibility of incorporating it into existing combination therapies (Spencer et al., 2016).

Given its demonstrated ability to cross the BBB (Di et al., 2016), MRZ was also evaluated for glioma therapy. In WHO grade IV glioma at first or second recurrence, MRZ was investigated alone and in combination with bevacizumab, yielding ORRs ranging from 3% to 50% across dose levels, though all cohorts were terminated early for tumor progression. While treatment-related adverse events occurred in all patients, DLTs were infrequent, and overall tolerability remained acceptable. However, a durable clinical benefit was not achieved. Subsequently, MRZ was tested in newly diagnosed glioblastoma in combination with standard temozolomide plus radiotherapy. Dose escalation identified 0.8 mg/m^2^ as the recommended phase II dose. The most common adverse events were fatigue, nausea, hallucinations, and vomiting, whereas grade ≥3 CNS toxicities (principally reversible ataxia and confusion) were mitigated by dose reduction. Among 66 patients treated at the recommended dose, median OS reached 14.8 months (NCT02330562) (Clinicaltrials.Gov, 2015), comparable to standard therapy (Stupp et al., 2005). An international phase III trial followed, but MRZ (0.8 mg/m^2^) addition failed to significantly improve PFS (6.34 vs. 6.11 months) and increased grade ≥3 adverse events (37% vs. 27%). Furthermore, Health-related Quality of Life scores and Mini Mental State Examination performance deteriorated more in the experimental arm, reflecting persistent neurotoxicity at larger scale (NCT03345095) (Clinicaltrials.Gov, 2018).

Beyond MM and glioma, MRZ showed minimal efficacy in other malignancies. In a phase I study of advanced cancers (NCT00629473), only one of 14 lymphoma patients achieved a complete response lasting ≥10 cycles, while no objective responses were observed among 24 patients with solid tumors, and only one of three leukemia patients maintained stable disease (Harrison et al., 2016).

The disappointing clinical performance of marizomib, both as monotherapy and in combination regimens, has tempered enthusiasm for its further development. This likely explains why the phase I trial evaluating MRZ plus the histone deacetylase inhibitor vorinostat in non-small-cell lung, pancreatic, melanoma, or lymphoma patients remains completed but undisclosed (Clinicaltrials.Gov, 2008), and why subsequent studies in recurrent low-grade and anaplastic ependymoma, pediatric diffuse intrinsic pontine glioma combined with panobinostat, and other CNS malignancies have all been terminated (Clinicaltrials.Gov, 2020a; Clinicaltrials.Gov, 2020b; Clinicaltrials.Gov, 2024). At present, no active marizomib-sponsored clinical trials are ongoing.

Compared with bortezomib (BTZ), carfilzomib (CFZ), and ixazomib, marizomib (MRZ) has demonstrated inferior clinical efficacy, which underscores that the robust preclinical activity of natural products does not necessarily guarantee successful clinical translation. MRZ exhibited potent nanomolar inhibitory activity against the proteasome β5 subunit *in vitro*, which propelled its advancement into subsequent clinical trials. However, its poor selectivity, characterized by strong inhibitory effects on both the β1 and β2 subunits, renders it prone to damaging neurons in the central nervous system that are highly dependent on protein homeostasis and extremely sensitive to sustained broad-spectrum proteasome inhibition. Neurotoxicity can only be avoided at extremely low doses, and the risk of central nervous system-related adverse events rises sharply with even a small increase in dosage. Although the relatively high lipophilicity of MRZ confers BBB penetration, it also facilitates non-specific binding to brain tissue, resulting in a significantly lower free drug concentration in the brain than the total drug concentration. Furthermore, compared with hematological malignancies such as MM, glioma possesses lower intrinsic sensitivity to proteasome inhibition. Consequently, the neurotoxicity threshold of MRZ is reached prior to the effective anti-tumor therapeutic dose, making it difficult to achieve the therapeutic concentrations required for glioma within a safe dosage range, ultimately leading to limited clinical benefit in the treatment of glioma. Nevertheless, this does not imply that all research on marizomib is valueless. Its oral bioavailability and BBB penetration still render it a proteasome inhibitor worthy of further development. Structural optimization to enhance its selectivity for the primary anti-tumor target, the β5 subunit, thereby improving the therapeutic index, is expected to become an important direction for the subsequent research and structural modification of MRZ.

## 6 Translational barriers for natural PIs

As discussed in previous sections, although several natural PIs have demonstrated promising antitumor activity in both *in vitro* and *in vivo* studies, no natural PI has yet been successfully approved for clinical use. The major translational barriers mainly lie in several aspects. First, poor pharmacokinetic properties represent a major limitation. Many natural PIs exhibit inadequate metabolic stability, short half-lives, poor cellular permeability, and low bioavailability. For example, lactacystin requires metabolic conversion *in vivo* to its active form, clasto-lactacystin β-lactone, which results in less predictable pharmacokinetics (Hitora and Tsukamoto, 2025); moreover, this active metabolite is chemically unstable in aqueous solutions. Second, toxicity and lack of selectivity also restrict their clinical development. Natural products often act on multiple molecular targets, which can lead to undesirable off-target effects (An et al., 2022). For instance, in addition to proteasome inhibition, celastrol has been reported to modulate several signaling pathways, including NF-κB signaling, NADPH oxidases, NEDD4 (Hong et al., 2023), and FXR, and inhibition of FXR can lead to intestinal bleeding (Dai et al., 2023). Third, structural complexity poses additional challenges. Many natural products possess highly complex structures, making their extraction or total synthesis costly and technically demanding, which further limits large-scale drug development.

Taken together, these challenges have so far prevented the successful clinical translation of natural PIs. To date, marizomib is the only natural PI that has entered clinical trials, although its development has encountered difficulties, including limited efficacy in solid tumors, which has hindered further progress. Nevertheless, natural products remain valuable lead compounds for drug discovery and provide important structural scaffolds for the development of improved proteasome inhibitors. A notable example is epoxomicin, whose suboptimal pharmacokinetic properties prompted medicinal chemistry optimization, ultimately leading to the development of the clinically approved drug carfilzomib (Kim and Crews, 2013).

## 7 Future perspectives

Following the sequential introduction of bortezomib (2003), carfilzomib (2012), and ixazomib (Ixa, 2015) into the clinic (Fricker, 2020), proteasome inhibition has become the cornerstone of first- and second-line therapy for multiple myeloma. However, the dose-dependent peripheral neuropathy associated with bortezomib (Manasanch and Orlowski, 2017), the severe cardiovascular toxicity of carfilzomib (Georgios et al., 2023), as well as cytopenias and gastrointestinal adverse events of ixazomib (Alrasheed et al., 2024) have substantially limited their therapy. In addition, although PIs have achieved remarkable success in the treatment of hematological malignancies such as multiple myeloma and mantle cell lymphoma, their clinical application continues to face challenges including primary and acquired resistance and limited efficacy in solid tumors.

Solid tumor cells typically produce fewer secreted proteins and experience lower basal proteotoxic stress. Consequently, they rely less on proteasome-mediated protein degradation and generally exhibit lower levels of immunoproteasome expression, which may contribute to their reduced sensitivity to proteasome inhibitors compared with multiple myeloma cells. In addition, the tumor microenvironment of solid tumor leads to poor drug penetration of PIs, resulting in limited efficacy in clinical trials (Fanucchi et al., 2006; Kontopodis et al., 2016). Activation of angiogenic signaling pathways is a key driver of tumor progression and therapeutic resistance in solid tumors (Li et al., 2023; Liu et al., 2024; Zhou et al., 2022; Cheng et al., 2021). PIs are implicated in the regulation of angiogenesis, and clinical trials in solid tumors have evaluated the combination of PIs with anti-angiogenic agents, demonstrating evidence of therapeutic benefit (NCT00428545) (Falchook et al., 2014).

Future research should focus on elucidating the molecular mechanisms underlying resistance. As discussed above, combination strategies targeting proteostasis networks–for example, combining proteasome inhibitors with HSP90 inhibitors or histone deacetylase inhibitors–have already entered clinical trials. In addition, despite the catalytic proteasome-dependent resistance mechanisms, non-catalytic proteasome targeting, particularly strategies directed at the 19S regulatory particle, has emerged as an important research direction in the field of proteasome inhibitor development. Unlike conventional proteasome inhibitors that target the β5 catalytic site of the 20S core particle and are therefore susceptible to resistance mechanisms such as PSMB5 mutations, targeting the 19S regulatory particle represents a fundamentally different approach. This strategy does not directly target the catalytic degradation of proteins within the proteasome; instead, it functions through the upstream steps, such as deubiquitination, thereby avoiding resistance mechanisms associated with conventional catalytic proteasome inhibitors. To date, several small-molecule inhibitors targeting components of the 19S regulatory particle, including RPN11 (Li et al., 2017), USP14 (Wang F. et al., 2021), and UCHL5 (Han et al., 2026), have been reported. In addition, because proteasome activity is not only determined by the catalytic sites but also regulated by conformational dynamics of the proteasome complex, allosteric modulation strategies are also worth further investigation. Low serum sodium levels (hyponatremia) have been reported to correlate with chemoresistance in cancer and are also a common adverse event in bortezomib-treated patients (Paydas, 2012; An et al., 2026); therefore, investigating the potential association between hyponatremia and bortezomib resistance represents an important direction for future research. Concurrently, optimizing dosing regimens and developing next-generation inhibitors with improved selectivity and safety profiles are expected to address the limitations of existing drugs. Beyond the proteasome itself, emerging therapeutic approaches are expanding to upstream regulators of the ubiquitin–proteasome pathway, such as deubiquitinating enzyme and E3 ligase inhibitors, as well as novel protein degradation technologies like proteolysis-targeting chimeras (PROTACs). The application of CAR-T therapy in MM continues to evolve (Ding and Wu, 2024; Xiang X. R. et al., 2020; Zhang et al., 2023a). In addition, novel anti-myeloma targets, including LILRB1, COMMD3, and SAE1, are being actively explored in MM (Liu et al., 2025; Sun et al., 2016; Tao et al., 2021; Wang Y. J. et al., 2025; Zhang et al., 2025; Xian et al., 2024). These strategies may act synergistically with PIs to enhance antitumor efficacy (Li J. et al., 2025; Xian et al., 2017). In addition, addressing PI-associated toxicities, such as neurotoxicity, cardiotoxicity, and gastrointestinal adverse effects, through optimized clinical monitoring and rational combination strategies represents an important direction for future research (Li et al., 2024). Ultimately, integrating biomarker-guided patient stratification, individualized therapy, and rational drug combinations will maximize the therapeutic potential of proteasome inhibition, extending its application from hematologic cancers to solid tumors.

Among the numerous efforts to improve the clinical utility of PIs, increasing attention has been directed toward structurally diverse natural products as potential sources of next-generation inhibitors with enhanced efficacy and reduced toxicity. Among these, several classes—including β-lactones, peptidic epoxyketones, and macrolides/macrolactams—have been extensively studied, yielding many well-characterized natural PIs. In addition, numerous other compounds that fall outside these categories also exhibit proteasome-inhibitory activity. Their potencies range from the nanomolar to micromolar scale, providing a broad array of scaffolds for further structural optimization and drug discovery. Many of these natural products are also multi-target agents, capable of modulating diverse molecular pathways. Identifying such multitarget inhibitors for specific diseases may offer valuable lead structures for future drug development. At present, marizomib remains the only natural product-derived proteasome inhibitor that has advanced to clinical trials. Its oral bioavailability and ability to cross the BBB have led to its approval for the clinical trials of glioblastoma and myeloma with central nervous system involvement. Unfortunately, the current clinical trial outcomes of marizomib did not provide enough evidence for its benefit in patients, suggesting that further structural optimization may be required to improve its pharmacokinetic properties and therapeutic efficacy. With the rapid advancement of artificial intelligence (AI) and computational technologies, future research can leverage machine learning and molecular simulations to provide more targeted guidance for the structural modification of natural products, as well as to enable virtual screening for the discovery of low-toxicity, high-efficiency candidate compounds.

Beyond compounds that directly target the proteasome, numerous natural products have been identified that enhance the efficacy of PIs through distinct mechanisms. These findings provide promising entry points for overcoming drug resistance or improving sensitivity to proteasome inhibition. Future research may therefore focus on exploring combination strategies based on these natural products to sensitize tumors to PIs or counteract resistance. Moreover, elucidating the mechanistic basis of how these compounds potentiate proteasome inhibition may uncover key regulatory targets that govern sensitivity and resistance, thereby offering theoretical and practical foundations for rational drug design and therapeutic optimization.

Overall, the development of natural product-derived PIs and sensitizers has continued to advance over the past several decades. To date, only marizomib has entered clinical trials—the slow progress in research and development is likely because existing PIs already substantially prolong the survival of patients with multiple myeloma. Nevertheless, for patients with primary or acquired resistance, further development of novel PIs with distinct chemical scaffolds or rational combination regimens capable of overcoming resistance remains of great significance. Indeed, since the introduction of PIs and immunomodulatory drugs, multiple myeloma has gradually evolved from a fatal disease into a manageable chronic condition. Accelerating this transformation will depend on the discovery of new agents that can effectively circumvent drug resistance. In addition, PIs with new structural frameworks hold great promise for extending the therapeutic potential of this class to solid tumors. The vast structural diversity of natural products provides an abundant reservoir for drug discovery, underscoring the importance of continued exploration and innovation in this area.
