# Supplementary material for: Targeting the proteasome in cancer therapy: development and future opportunities in natural products
Source: Front Pharmacol. 2026 May 4;17:1806787. doi: 10.3389/fphar.2026.1806787 (PMC13180746; doi:10.3389/fphar.2026.1806787)
Supplement: Supplementary file 1 [file Supplementaryfile1.pdf]

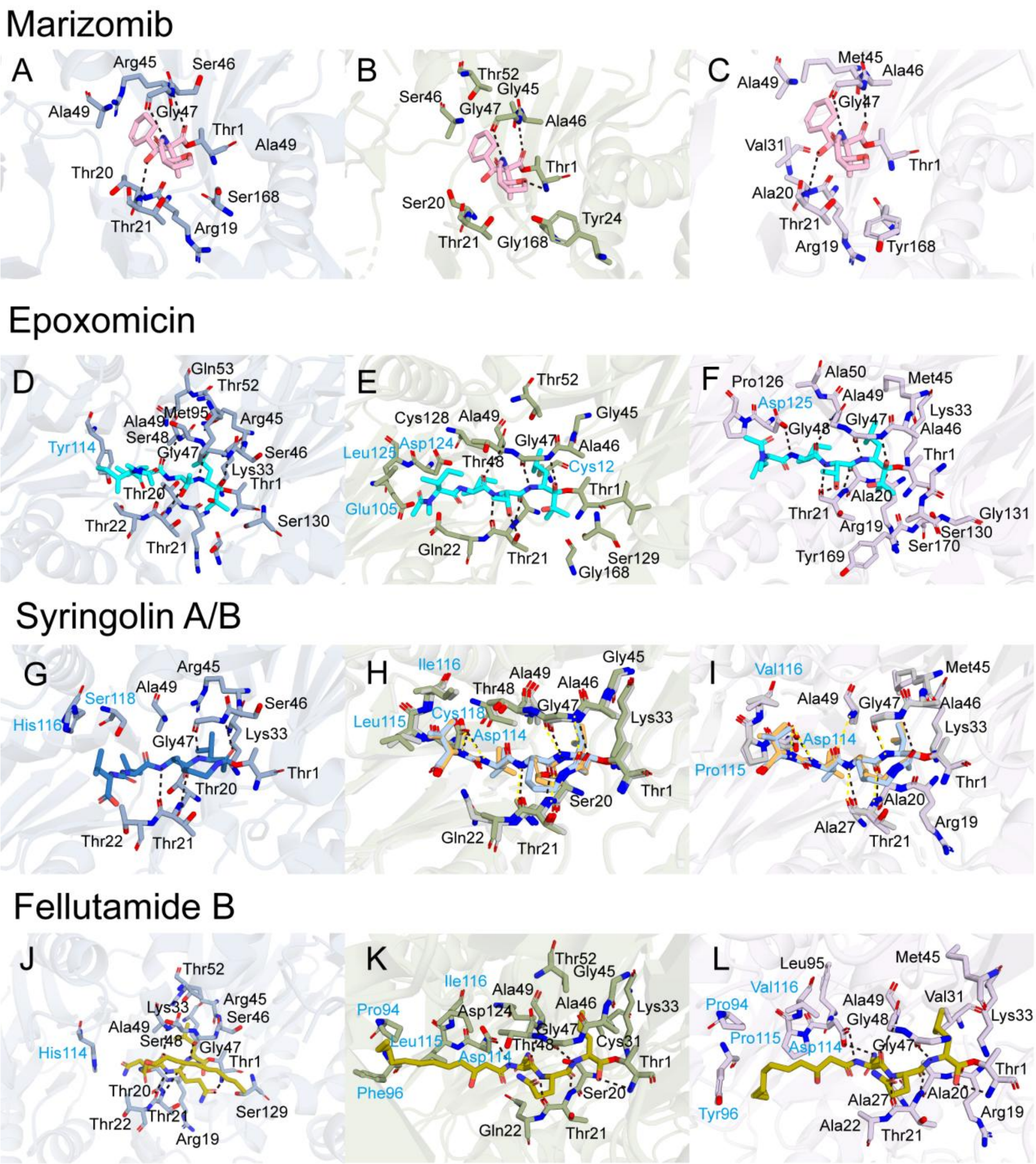

Figure S1. Structural basis for the selectivity of distinct proteasome inhibitors toward 20S proteasome catalytic subunits

Marizomib (A–C) (PDB: 2FAK), epoxomicin (D–F) (PDB: 5LF0), syringolin A/B (G–I) (PDB: 2ZCY, 3GPJ), and fellutamide B (J–L) (PDB: 3D29) target the proteasome β1 (pale blue), β2 (beige-green), and β5 (lilac) subunits as indicated. Pocket-forming residues from adjacent β subunits are colored blue. In H, syringolin A is shown in orange, with its interacting residues in gray and hydrogen bonds in black; syringolin B is shown in light blue, with its interacting residues in green and hydrogen bonds in yellow. In I, syringolin A/B is colored the same as in H, while the interacting residues of syringolin B are shown in lilac.
